# Addition and Oxidation Reactivity of a Pentacoordinate Nickelacyclobutane

**DOI:** 10.1002/chem.202404133

**Published:** 2025-01-08

**Authors:** María L. G. Sansores‐Paredes, Martin Lutz, Marc‐Etienne Moret

**Affiliations:** ^1^ Organic Chemistry and Catalysis Faculty of Science Utrecht University Institute for Sustainable and Circular Chemistry Universitetisweg 99 3584 CG Utrecht, The Netherlands; ^2^ Structural Biochemistry Faculty of Science Utrecht University Bijvoet Centre for Biomolecular Research Universitetisweg 99 3584 CG Utrecht, The Netherlands

**Keywords:** Nickelacyclobutane, Protonolysis, σ-bond metathesis, Bond activation, Organometallic reaction mechanisms

## Abstract

Nickelacyclobutanes are reactive intermediates in catalytic cycles including cyclopropanation and insertion reactions. The stoichiometric study of these intermediates has shown that their reactivity is highly influenced by the coordination environment of the nickel center. A pentacoordinated nickelacyclobutane embedded in a diphosphine pincer ligand has been shown to selectively undergo various reactions with exogenous ligands, including [2+2] cycloreversion and carbene transfer to an isocyanide. Herein, we investigate the reactivity of the pentacoordinated nickelacyclobutane towards addition and oxidation reactions. Addition reactions lead to ring‐opening to form stable square planar Ni(II) compounds, while metal oxidation enhances [2+2] cycloreversion. DFT calculations are used to shed light on the different mechanisms.

## Graphical abstract

The reactivity of a pentacoordinate nickelacyclobutane following addition and one‐electron oxidation pathways is described. Ring‐opening products are observed with substrates containing a stabilizing counteranion. Pathways leading to an oxidized nickelacyclobutane yield [2+2] cycloreversion‐type products, suggesting that this pathway is accessible to oxidized nickelacyclobutanes.

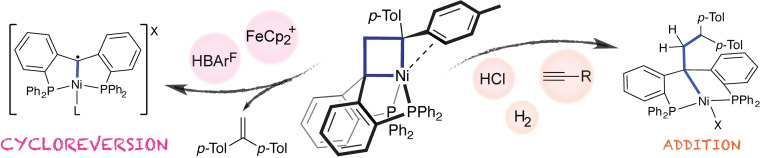

## Introduction

In the transition towards more sustainable chemistry, catalysis using nickel complexes is attracting great interest, because of nickel's abundance and relative low toxicity.^1−3^ A clear understanding of the nature of the intermediates involved in these processes is key to unleash their full potential and achieve selective chemical transformations.

**Scheme 1:**
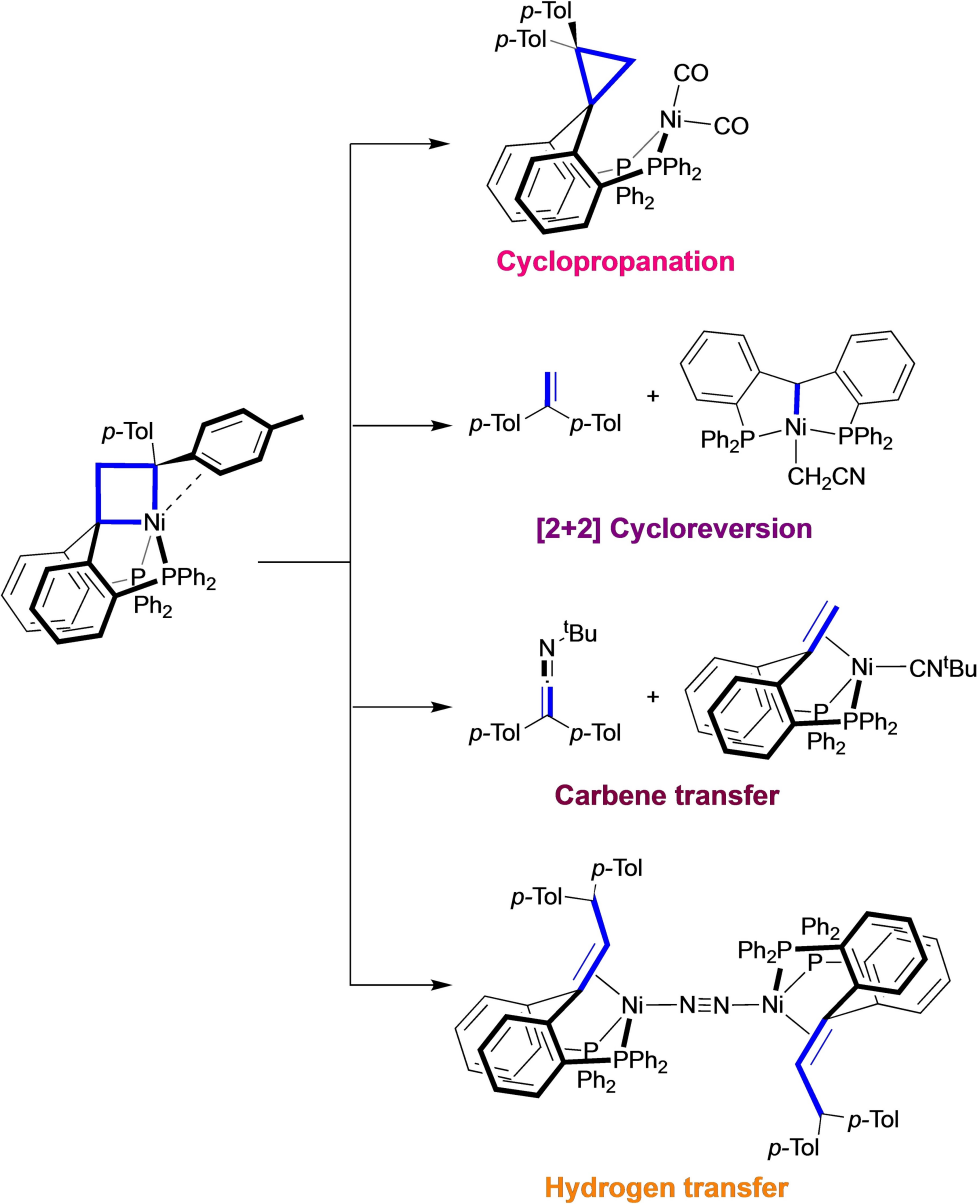
Reactivity of a pentacoordinated nickelacyclobutane.^12,16^

For instance, the study of nickelacyclobutanes, mainly known as intermediates in catalytic cyclopropanation,^4−11^ has shown that their coordination number and electronic structure is crucial to enhance and open new reactive pathways.^12^ In contrast with previously investigated square planar structures,^6,13−15^ the pentacoordinate nickelacyclobutane can undergo selective and unusual reactions induced by exogenous ligands/reagents (Scheme 1):^12,16^ cyclopropanation (with CO), [2+2] cycloreversion (with MeCN), carbene transfer (with CN^t^Bu), and “hydrogen transfer” yielding an olefin Ni(0) complex in benzene and toluene solutions. The stability provided by the diphosphine pincer framework makes the pentacoordinated nickelacyclobutane an excellent model compound to study the reactivity of this class of intermediates.

In this contribution, we complement our previous studies on the reactivity of the pentacoordinated nickelacyclobutane, by investigating addition and oxidation reactions. Two main pathways are observed: reactivity towards substrates such as H_2_, HCl, and terminal alkynes shows X−H (X= H, Cl or C) activation and concomitant ring opening to yield stable Ni(II) square planar structures. Remarkably, one‐electron chemical oxidation and exposure to Brookhart's acid lead to [2+2] cycloreversion products. DFT calculations shed light on the possible mechanisms involving σ‐metathesis or oxidative addition processes.

## Results and Discussion

### Activation of the H−H Bond

**Scheme 2:**
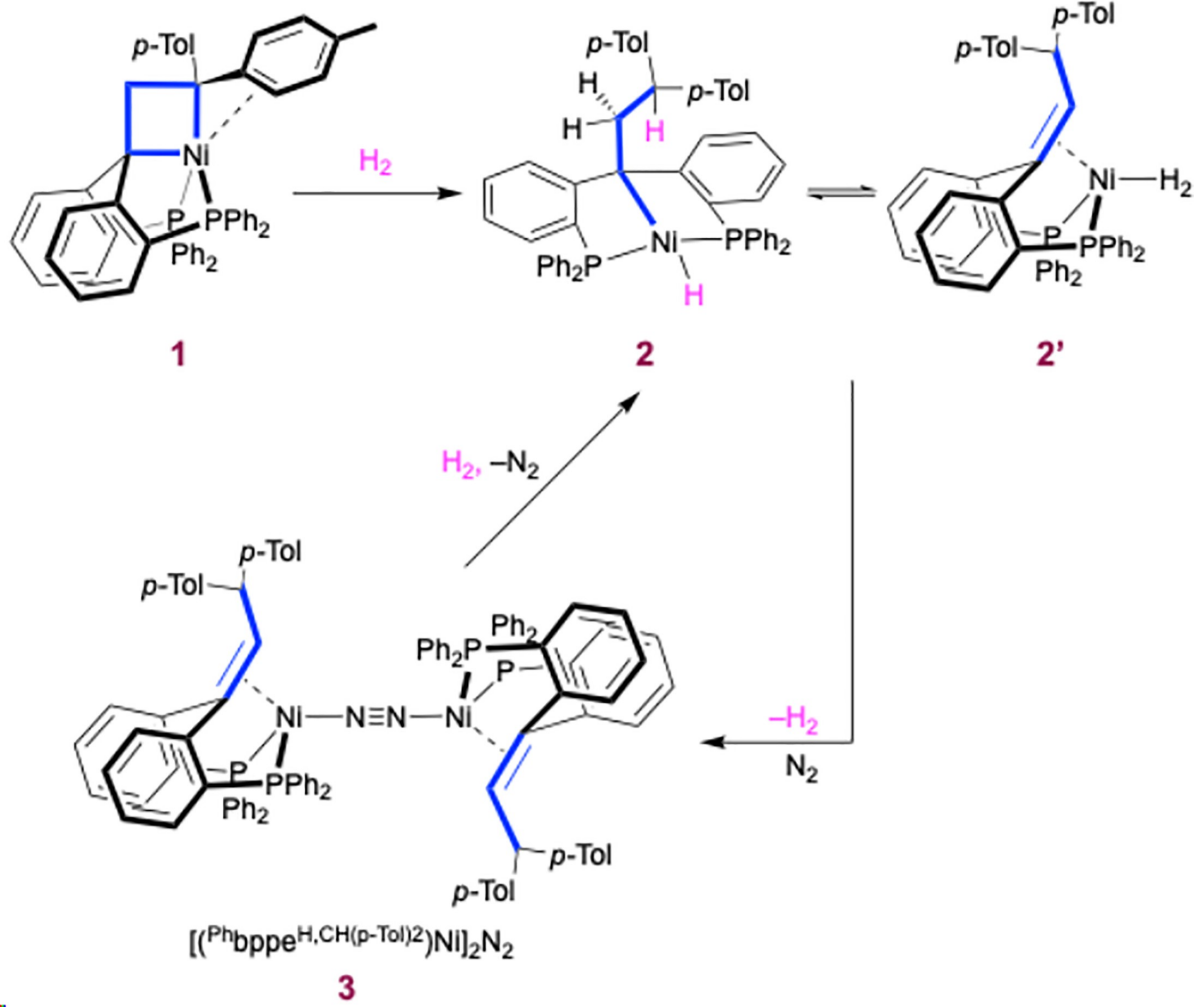
Reactivity of nickelacyclobutane with H_2_.

We first investigated the reactivity towards molecules presenting covalent bonds such as H_2_. Exposing a d^8^‐toluene solution of nickelacyclobutane **1** to 1 atm of H_2_ immediately affords a mixture of species (Scheme 2). Analysis by ^1^H NMR at 25 °C showed broad features and no resonance of H_2_ was located. The corresponding ^31^P{^1^H} NMR spectrum showed a broad peak at 40.1 ppm. NMR analysis at –40 °C showed a mixture of species in exchange with dissolved H_2_, where the major component was identified as the (alkyl)nickel hydrido complex **2** in equilibrium with a small amount of complex **2’**. Removal of the H_2_ atmosphere by freeze‐pump‐thaw and reintroduction of the N_2_ atmosphere resulted in the formation of **3 [(^Ph^bppe^H,CH(p−Tol)2^)Ni_2_]N_2_**. We had previously described the formation of **2** in equilibrium with **2’** by reacting complex **3** with H_2_ (1 atm) in more detail.^17^ Even though complex **1** thermally evolves to **3**,^12^ this transformation takes more than 24 h, which rules out complex **3** as an intermediate in this reaction.^12^

The observed Ni–C bond hydrogenolysis parallels previous reports by Miyashita, in which a bis(triphenylphosphine) nickelacyclobutane yielded alkyl chains after exposure to H_2_ under pyrolysis conditions (–50 to 50 °C) or in a H_2_/CO mixture.^18^ The stability provided by the PCP ligand framework now allows the observation of the ring‐opening reaction as a complete and selective reaction.

### Reactivity with Alkynes

**Figure 1:**
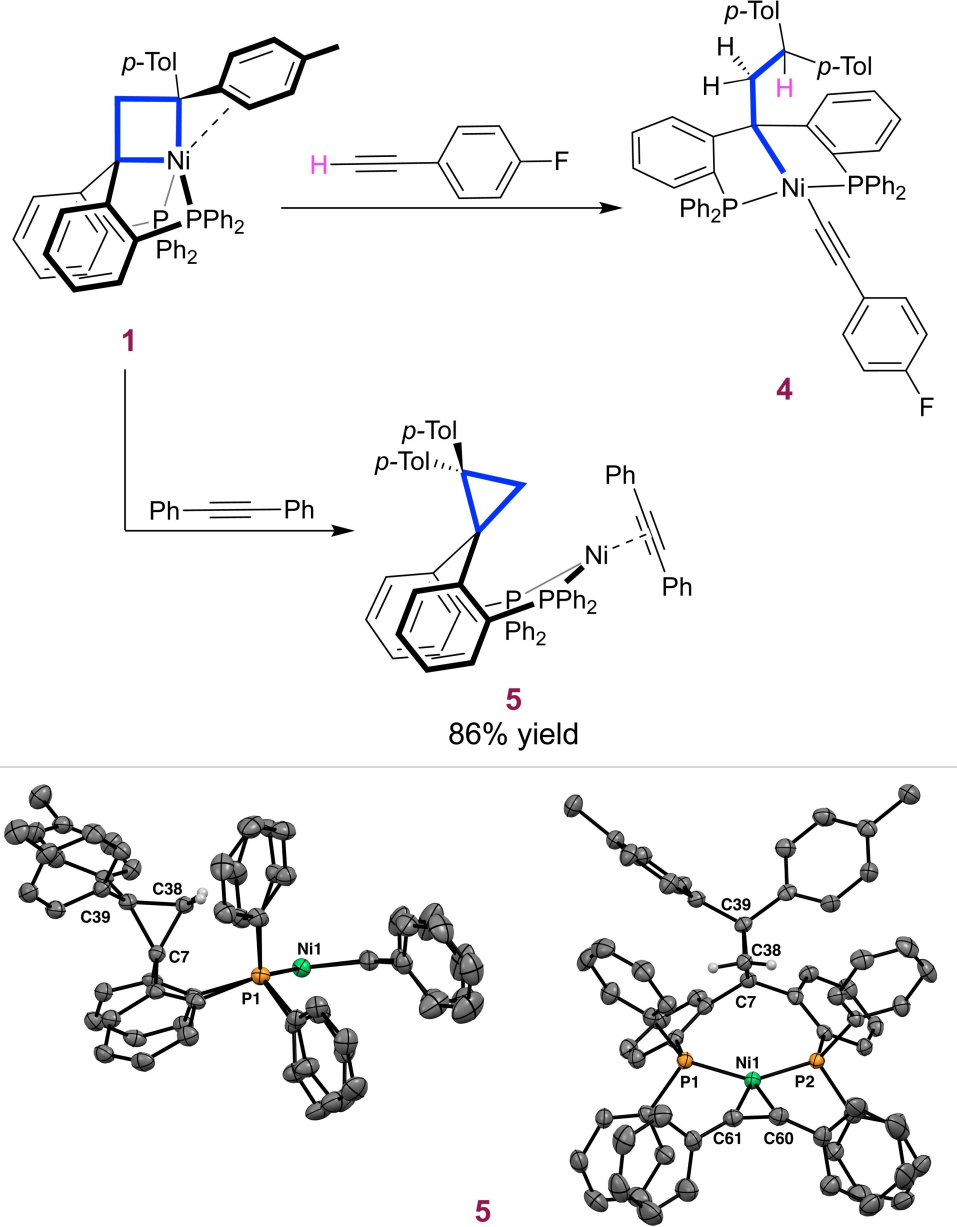
Top: reactivity of nickelacyclobutane **1** with terminal and internal alkynes. Bottom: two views of the molecular structure of **5** in the crystal. Displacement ellipsoids are drawn at the 50 % probability level. Solvent molecules and most H atoms are omitted for clarity. Selected bond lengths (Å): Ni1–P1 2.1494(7), Ni1–P2 2.1601(7), Ni1–C60 1.898(3), Ni1–C61 1.911(3), C60–C61 1.279(4), C7–C38 1.503(3), C7–C39 1.590(3), C38–C39 1.506(4).^25^

To continue to study the reactivity of the nickelacyclobutane **1**, we turned to the terminal alkyne 1‐ethynyl‐4‐fluorobenzene. Metallacycles are known to undergo migratory insertion with unsaturated molecules such as alkynes, CO, and isocyanides leading to ring enlargement, a pathway that could be used for the synthesis of other target products.^14,19−23^ Somewhat surprisingly, addition of 1.2 equivalents of 1‐ethynyl‐4‐fluorobenzene to nickelacyclobutane **1** leads instead to a C−H activation/ring opening product after 10 min (Figure 1). Complex **4** is identified as a previously described^24^ Ni(II) square planar species with the alkyl backbone and an alkynyl group as anionic ligands. Interestingly, complex **4** had also been observed when exposing the olefin complex **3** to 1‐ethynyl‐4‐fluorobenzene featuring identical spectroscopic features in C_6_D_6_.^24^

Next, we explored the reactivity of **1** with 1.1 equivalents of diphenylacetylene (DPA), an internal alkyne that could not undergo the same C−H activation process. The reaction is slower (24 h to completion) and affords complex **5** as the sole product (Figure 1). A single ^31^P{^1^H} NMR signal in C_6_D_6_ at 31.8 ppm indicates a symmetrical structure. In the ^1^H NMR spectrum, the CH_2_ of the backbone gives rise to a singlet at 3.74 ppm (see SI, section 3). In contrast with the anticipated insertion reaction, X‐ray diffraction analysis revealed that the nickelacyclobutane has undergone reductive elimination to generate a Ni(0) alkyne complex and a pendant cyclopropane ring (Figure 1). ^25^ The ligand structure and coordination mode in complex **5** are analogous to those in the corresponding previously reported Ni(CO)_2_ complex obtained by reaction of **1** with an excess of CO.^12^ This result corroborates the notion that π‐acceptor ligands can induce reductive elimination of the nickelacyclobutane to form a cyclopropane ring.

### Outer Sphere Oxidation

**Scheme 3:**
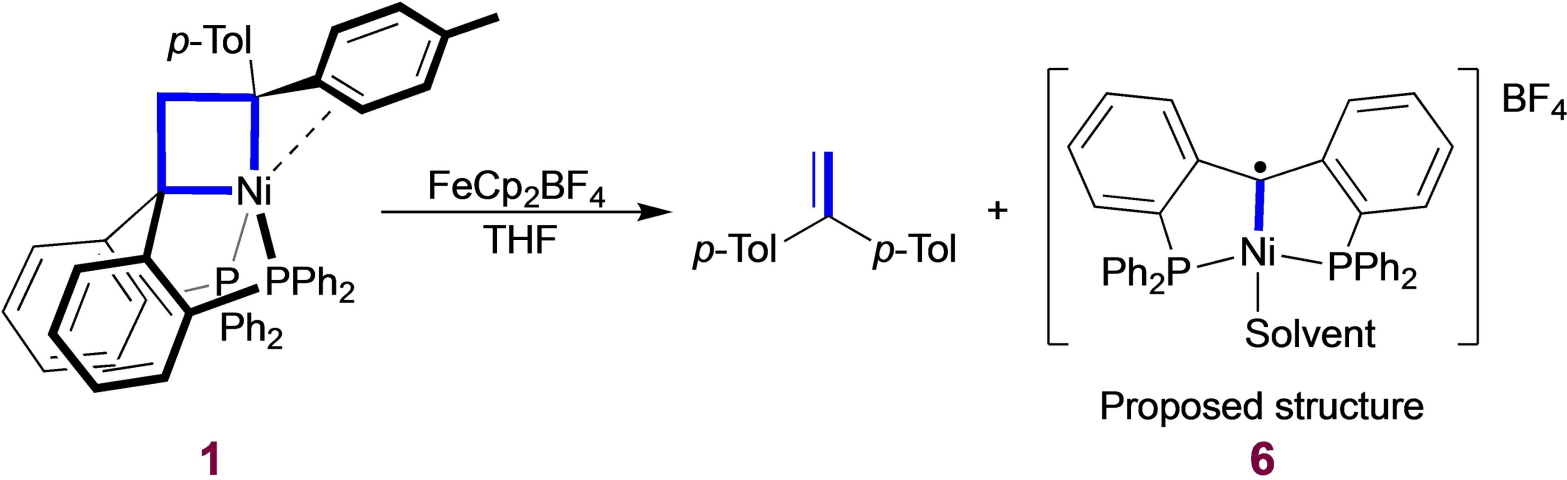
One‐electron oxidation reactivity of nickelacyclobutane **1**.

Oxidation reactions have been used to induce reactivity and give insight into how the electronic configuration affects the metal.^15,26−28^ The reaction in d^8^‐THF between nickelacyclobutane **1** and 1 equivalent of the one‐electron oxidant [FeCp_2_][BF_4_] yielded 1 equivalent of 1,1‐di(*p*‐tolyl)ethylene, a [2+2] cycloreversion product (Scheme 3), together with a paramagnetic product. Full conversion to the olefin is observed by ^1^H NMR after 10 min (see SI, section 2). The paramagnetic product (**6**) decomposes shortly after crude isolation by precipitation with hexane, preventing the obtention of clean NMR data. The isolated samples nevertheless gave rise to an intense EPR signal at 298 K and 90 K with a g‐value (∼2.0) consistent with an organic radical (see SI, section 3). These observations are consistent with an oxidized Ni(III) nickelacyclobutane undergoing rapid [2+2] cycloreversion to form a formally Ni(I) square planar (PC_carbene_P)Ni complex **6** and 1,1‐di(*p*‐tolyl)ethylene. On the basis of related literature reports (see SI, section 2), the formally Ni(I) carbene is likely better described as a square‐planar Ni(II) complex bearing an alkyl radical (Scheme 3), in agreement with the EPR spectrum.^29−31^ In support of this interpretation, geometry optimization of **6** (using THF as solvent molecule) with doublet multiplicity led to the proposed organic radical (see SI, section 5). This reactivity differs from previous reports for nickelacyclobutanes, in which the oxidation of a bis(triphenylphosphine) nickelacyclobutane with Ce(IV) and O_2_ led to the observation of cyclopropanation products.^15^ Understanding this chemical transformation could be of interest for the development of olefin metathesis catalysts based on nickel or other base metals.

### Reactions with Brønsted Acids

**Figure 2:**
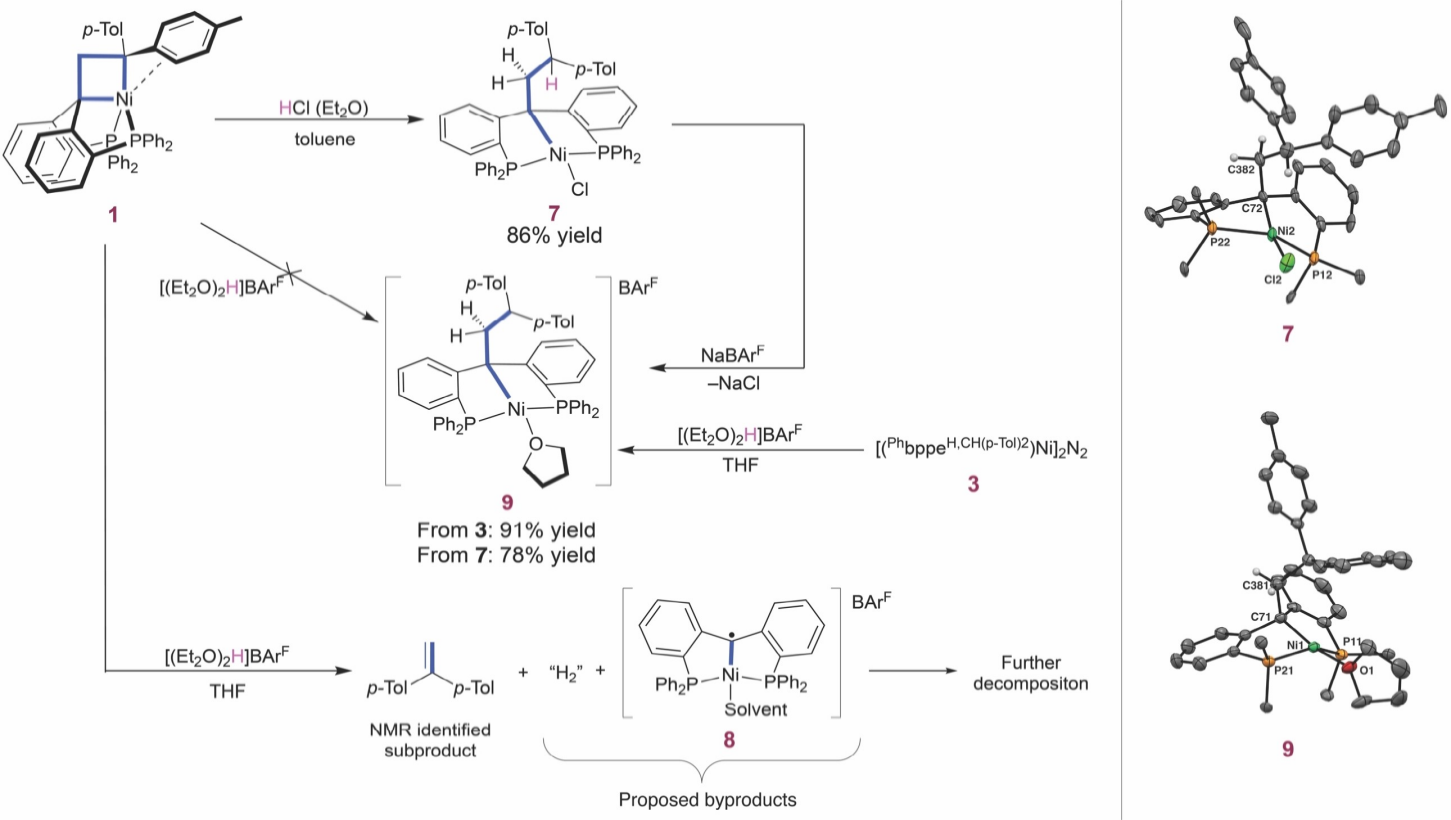
On the left: reactivity of nickelacyclobutane **1** with Brønsted acids and alternative synthesis of product **9**. On the right: molecular structure of **7** and **9**. Displacement ellipsoids are drawn at the 50 % probability level. Solvent molecules, most H atoms and phenyl rings from the phosphines are omitted for clarity. For **7**, only one of the two independent molecules is shown. Selected bond lengths (Å) and angles (°), for **7**: Ni2–P22 2.1853(16), Ni2–P12 2.1562(15), Ni2–Cl2 2.2245(18), Ni2–C72 2.031(6), C72–C382 1.539(7), P12–Ni2–P22 145.87(7), C72–Ni2–Cl2 165.75(16), C72–Ni2–P12 86.35(16), P12–Ni2–Cl2 98.97(7), P22–Ni2–Cl2 97.72(7), P22–Ni2–C72 84.67(15). For **9**: Ni1–P21 2.1949(6), Ni1–P11 2.1624(7), Ni–O1 1.9974(16), Ni1–C71 1.998(2), C71–C381 1.560(3), P11–Ni1–P12 146.43(3), O1–Ni1–C71 168.24(8), P21–Ni1–C71 86.33(7), C71–Ni1–P11 86.32(7), P11–Ni1–O1 98.41(5), O1–Ni1–P21 95.33(5).^25^

Protonolysis of M−C bonds is a subject of ongoing research because mechanistic subtleties, e. g. the distinction between an oxidative addition/reductive elimination sequence and concerted σ‐bond metathesis, are highly relevant for the microscopic reverse C−H bond activation reactions.^32−35^ Classically, addition of acids, such as HCl, has also been used as a way to identify the presence of nickelacyclobutanes by forming an alkane chain and a derived NiX_2_ complex.^15,32,37−41^ Addition of approximately 1.1 equivalents of HCl dissolved in Et_2_O to nickelacyclobutane **1** yielded the square planar complex **7** (Figure 2). One Ni–C bond has been cleaved, a new Ni–Cl bond has been formed, and the proton has been transferred to the α‐carbon bearing two *p*‐tolyl substituents. The alkyl CH_2_ and CH protons in **7** give rise to ^1^H NMR(C_6_D_6_) signals at 2.77 ppm (d, *J*_*H,H*_=6.8 Hz, 2H) and 5.15 ppm (t, *J*_*H,H*_=6.6 Hz, 1H), respectively. Interestingly, the CH signal is shifted downfield, which suggests a possible anagostic interaction with the nickel center.^42^ The ^31^P{^1^H} NMR spectrum of **7** shows a singlet at 24.9 ppm suggesting that both phosphorus atoms are equivalent by symmetry. Protonolysis of the remaining C−Ni bond was not observed, even when an excess of HCl was used. The resistance to protonolysis can be explained by the stability provided by the PCP pincer structure.

Crystals of **7** suitable for X‐ray diffraction were obtained by slow vapor diffusion of hexane into a concentrated toluene solution of **7** (Figure 2).^25^ Complex **7** presents a distorted square planar structure (Σ(Ni)= 366.9(2)°): the P12–Ni–P12 angle is more bent 145.87(7)° than the C72–Ni2–Cl2 angle 165.75(16)°. Moreover, the C72–C381 bond length of 1.539(7) Å confirms the single bond character of this bond.

To obtain more insight into the protonolysis of **1**, we used Brookhart's acid [(Et_2_O)_2_H]BAr^F^ (BAr^F^=tetrakis[(3,5‐trifluoromethyl)phenyl]borate) which, unlike HCl, contains a non‐coordinating anion. The reaction of **1** with 1.1 equivalents of Brookhart's acid in THF, (Figure 2), led to full conversion to 1,1‐di(*p*‐tolyl)ethylene and a paramagnetic mixture containing the BAr^F^ anion detected by ^19^F NMR at –63.42 ppm (see SI section 2.2). Reasoning that a metal‐centered protonation of the nickelacyclobutane would lead to the formation of highly oxidized intermediates enhancing the [2+2] cycloreversion pathway, the paramagnetic nickel‐containing product (**8**) is proposed to be an analogous structure to complex **6** and proposed as the intermediate species (see SI, section 2.2), but alternative (decomposition) products cannot be excluded. To maintain a correct mass balance, H_2_ is proposed as a byproduct but could not be detected by ^1^H NMR. Additionally, no products related to the protonation of the α‐carbon were observed (see SI, section 2.2).

Attempted isolation of **8** by precipitation with hexane led to an unstable pink oil which decomposed rapidly, preventing the growth of crystals suitable for X‐ray diffraction. An EPR spectrum of this isolated oil presented a weak signal that could not be straightforwardly interpreted.^25^ A single ^19^F NMR signal at –63.39 ppm in d^8^‐THF, corresponding to the (slightly paramagnetically shifted) BAr^F^ anion was observed. The corresponding ^1^H NMR in d^8^‐THF showed broad features in the 9.5‐16.5 ppm region, which resemble those observed in the oxidation reaction to complex **6** (Scheme 3).

Even though the identification of the nickel‐based products is not fully conclusive, the presence of 1,1‐di(*p*‐tolyl)ethylene seems consistent with initial metal protonation, where the oxidation of the metal would lead to [2+2] cycloreversion instead of the expected protonolysis product.

Nevertheless, this result is somewhat surprising: Brookhart's acid has been reported to be able to protonate metal‐olefin complexes and metallacycles to yield stable complexes and alkane chains.^42−46^ To exclude the possibility that 1,1‐di(*p*‐tolyl)ethylene could be formed *after* ring opening by protolysis, the cationic Ni(II) complex **9** that would be formed by the latter reaction was synthesized independently in two different ways (Figure 2). First, addition of 1 equivalent of Brookhart's acid to a THF solution containing the alkene complex [(^Ph^bppe^H,CHptol2^)Ni]_2_N_2_ (**3**) lead to compound **9**, in which one molecule of THF completes the coordination sphere. A ^31^P{^1^H} NMR spectrum of **9** in C_6_D_6_ shows a singlet at 23.3 ppm. In the ^1^H NMR spectrum in C_6_D_6_, the CH proton is found at 4.80 ppm (t, *J*_*H,H*_= 5.2 Hz) and the CH_2_ at 2.19 ppm (d, *J*_*H,H*_=5.8 Hz). Complex **9** could also be prepared by chloride abstraction from complex **7** with NaBAr^F^. X‐ray crystal structure determination confirmed the molecular structure of **9** (Figure 2).^25^ It also presents a distorted square planar structure (Σ(Ni) 366.39(12)°) similar to structure **7**, with the P11‐Ni1‐P12 angle more bent 146.43(3) than the O1‐Ni1‐C71 angle 168.24(8). The bond length of C71–C381 1.560(3) Å is characteristic for a C_sp3_‐C_sp3_ bond. The C−H bond in γ position is not bound to the Ni center. These results show that a cationic square planar structure resulting from putative ring opening of the nickelacyclobutane with Brookhart's acid is thermally stable, ruling out product instability as the reason for the difference in reactivity between HCl and Brookhart's acid. Instead, they suggest active involvement of one the counteranions in the protonolysis step.

### Computational Studies

**Figure 3:**
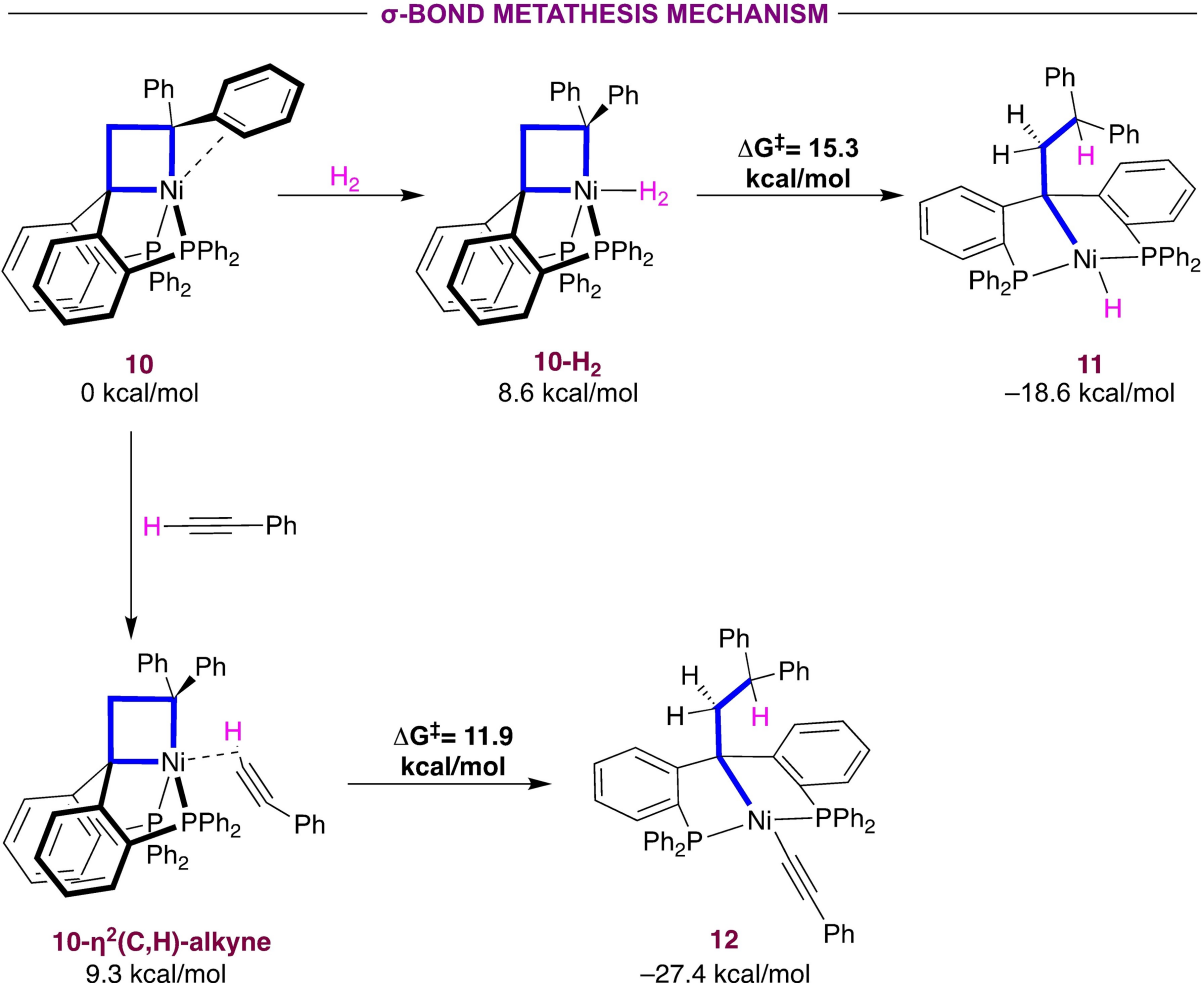
Gibbs energy for σ‐bond metathesis for the addition of H_2_ and phenylacetylene to **10**, calculated at the B3LYP‐GD3BJ/def2TZVP/SMD//B3LYP‐GD3BJ/6‐31g(d,p) level of theory. For H_2_, SMD= Toluene, for phenylacetylene SMD= C_6_H_6_.

**Figure 4:**
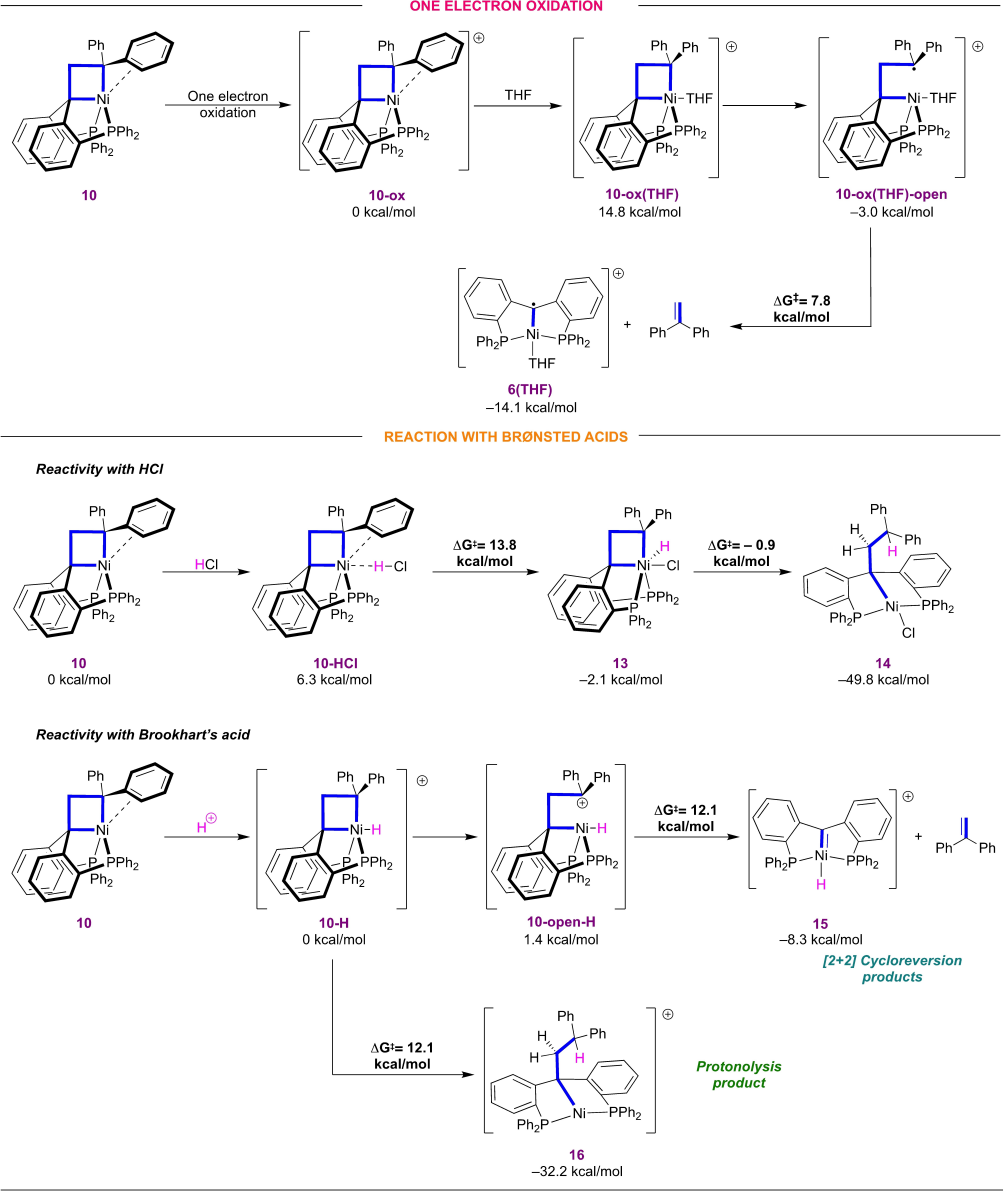
Gibbs energy for the one‐electron oxidation and protonation of nickelacyclobutane **10** calculated at the B3LYP‐GD3BJ/def2TZVP/SMD(THF)//B3LYP‐GD3BJ/6‐31g(d,p) level of theory.

To investigate mechanistic details of the reactions described above, DFT calculations were performed starting from the slightly truncated model **10** (Figure 3 and 4) obtained by substituting the tolyl substituents of the nickelacyclobutane by phenyl groups.^[47]^

### H−H and C−H Addition Reactions

Starting with the hydrogenolysis reaction of **1** with H_2_ (Figure 3), slightly endergonic coordination of H_2_ to nickel (+8.6 kcal/mol) is followed by a readily accessible σ‐bond metathesis transition state at ΔG^≠^=15.3 kcal/mol to yield the more stable (alkyl) nickel hydrido product **11** (–18.6 kcal/mol). Additionally, a Ni(IV) dihydride nickelacyclobutane was optimized but shown to be energetically prohibited at 24.3 kcal/mol (see SI, section 5.2). No other isomers of Ni(IV) dihydride were identified.

The σ‐bond metathesis mechanism is also favored for the addition of phenylacetylene (Figure 3). Starting with the coordination of phenylacetylene in η^2^(C,H) fashion (9.3 kcal/mol), the concerted transition state is readily available (ΔG^≠^=11.9 kcal/mol) yielding complex **12** (–27.4 kcal/mol). Initial coordination in η^2^(C,C) fashion is also feasible but only slightly more stable than the η^2^(C,H) mode (8.3 kcal/mol). In agreement with experiment, alternative reaction pathways were associated with higher activation barriers (see SI, section 5.3). The transition state for cyclopropanation starting from the η^2^(C,C) alkyne complex has a predicted energy barrier of 30.2 kcal/mol. In addition, the transition state for [1,2] migratory insertion to form a putative nickelacyclohexene was found higher in energy (ΔG^≠^=19.1 kcal/mol) compared with the C−H bond activation.

### Oxidation of the Nickelacyclobutane

Oxidation leads to the formation of a Ni(III) cyclobutane (**10‐ox**) intermediate (Figure 4), coordination of a THF molecule leads to the adduct **10‐ox(THF)** (14.8 kcal/mol). Homolytic cleavage of the Ni–C bond leads to a more stable organoradical Ni(II) structure with a low barrier (less than 3 kcal/mol) on the basis of a relaxed Potential Energy Surface scan (Figure S36). The resulting complex **10‐ox‐(THF)‐open** subsequently undergoes homolytic cleavage of the C−C bond of the backbone to form **6(THF)** and 1,1‐diphenylethylene (ΔG^≠^= 7.8 kcal/mol). A [2+2] cycloreversion route from **10‐ox** to yield (PC_carbene_P)Ni and 1,1‐diphenylethylene was computed with an overall barrier of ΔG^≠^= 17.8 kcal/mol (see SI, section 5.4). Both pathways present similar activation energies, but high concentration of THF used as solvent could enhance initial THF coordination. Overall, these results suggest that a different pathway on oxidized nickelacyclobutanes to yield [2+2] cycloreversion products via radical intermediates could be accessible.

### Reactivity with Brønsted Acids

Study of the protonolysis of square planar alkyl platinum(II) complexes have shown a good correlation between the nature of the HOMO and the type of protonolysis mechanism. If the HOMO is metal based, SEOX (oxidative addition) is enhanced. SE2 (concerted electrophilic displacement) can occur if the HOMO is the M−C sigma bond.^32−34^ The orbital analysis of nickelacyclobutane **10** was performed by extracting the HOMO and HOMO‐1 orbitals from the single point calculation at the B3LYP‐GD3BJ/def2TZVP level of theory (see SI, section 5.5). The HOMO is a metal centered orbital with delocalization along the pincer ligand. The σ‐bonding interaction of the key Ni–C bond is localized in the HOMO‐1 orbital, suggesting the pentacoordinate nickelacyclobutane is prone to undergo an oxidative protonation mechanism, in good agreement with the observed reactivity with Brookhart's acid.

For the study of the reaction with HCl, we explored both oxidative addition and σ‐bond metathesis processes. HCl in diethyl ether/toluene solution is reported to consist of a protonated diethyl ether molecule forming a contact ion‐pair with a chloride anion. In other words, the chloride anion remains closely associated with the proton and could assist protonation of the α‐carbon.^48^ To test this hypothesis, reactions of molecular HCl were computationally investigated. Formation of the **1‐HCl** adduct is readily accessible (6.3 kcal/mol) as a primarily electrostatic interaction that does not displace the phenyl substituent from the coordination sphere of nickel (Figure 4). For the oxidative addition pathway, four possible Ni(IV) structures were computed (see SI, section 5.5). An accessible pathway (ΔG^≠^=13.8 kcal/mol) was found to yield structure **13** (–2.1 kcal/mol), an octahedral Ni(IV) with the chloride anion trans to the Ni–C bond and the hydrogen in equatorial position. Promptly, **13** undergoes reductive elimination of the hydride and Cα yielding complex **14** (–49.8 kcal/mol). The transition state for σ‐bond metathesis is accessible but slightly higher in energy than the oxidative addition pathway (ΔG^≠^= 15.0 kcal/mol, see SI, section 5.5). Additionally, the [2+2] cycloreversion pathway from **10‐HCl** was found to be prohibitively high in energy (ΔG^≠^= 37.7 kcal/mol). These results suggest that oxidative addition of molecular HCl is a facile process for complex **10** and leads to the observed ring‐opening product. Alternatively, initial oxidative protonation followed by fast reaction with the nearby Cl^–^ anion could yield **13**, which could also explain why protonolysis with HCl affords different products than with HBAr^F^.

Next, we explored the oxidative protonation of the nickelacyclobutane to form a cationic Ni(IV) intermediate (Figure 4). Optimized structure **10‐H** shows a distorted trigonal pyramidal structure with the Ni–H bond slightly bent towards one of the phosphine arms (see SI, section 5.5). The triplet state structure was found to be energetically higher than the singlet state (11.2 kcal/mol).

From complex **10‐H**, hydride insertion to the Cα from is feasible *via* a low‐lying transition state (ΔG^≠^= 12.1 kcal/mol). and strongly exergonic (– 32.2 kcal/mol). The latter barrier sets an upper limit for the possible barrier for cycloreversion after protonation.

For the [2+2] cycloreversion pathway starting from **10‐H**, the first step is facile heterolytic cleavage of the Ni–C bond leading to reduction of nickel to Ni(II) and formation of a carbocation **10‐open‐H** (1.4 kcal/mol), in similarity with the oxidation pathway (See relaxed Potential Energy Surface scan, Figure S41). The transition state for C−C cleavage yielding cycloreversion products is readily accessible at ΔG^≠^= 12.1 kcal/mol. Additionally, other spin states of complex **10‐open‐H** were calculated, showing that **10‐open‐H** is the less energetic structure (see SI, section 5.5).

The transition state for cycloreversion and C−H reductive elimination are energetically comparable. Both pathways are predicted to be readily accessible from a putative Ni(IV)–H intermediate, which does not fully explain the experimentally observed preference for olefin extrusion. However, the electronic structure of **10‐open‐H** is likely too complex to be accurately accounted for by a single‐determinant DFT method, preventing an accurate prediction of the associated energy barriers. An additional mechanistic possibility is that the protonated Ni(IV)–H species **1‐H** would rapidly lose an H• radical – possibly forming H_2_ in a bimolecular fashion – to form a Ni(III) metallacycle (see SI, Scheme S5). The latter was shown to rapidly release the olefin fragment when formed by direct oxidation of **1** (see above). All in all, our DFT investigations cannot unambiguously identify the mechanism at play, but they show that several decomposition pathways compete within a narrow range of activation energies for oxidized nickelacyclobutanes.

## Conclusions

We have investigated the reactivity of a pentacoordinated nickelacyclobutane incorporated in a diphosphine pincer ligand towards addition and oxidation reactions. With reagents containing a reactive element–hydrogen bond (such as H_2,_ HCl, and 1‐ethynyl‐4‐fluorobenzene), the nickelacyclobutane undergoes ring opening reactions yielding stable Ni(II) square planar structures. These reactions likely proceed *via* a σ‐bond metathesis pathways with one of the Ni–C bonds, and DFT calculations identify low‐lying concerted transition states in all cases. In addition, chemical one‐electron oxidation generates the [2+2] cycloreversion product 1,1‐di(*p*‐tolyl)ethylene as the major organic product. The metal‐containing product is most likely a formally (PC_carbene_P)Ni(I) fragment that is better described as an organic radical bound to Ni(II) according to its EPR spectrum and literature precedents. DFT calculations support initial metal‐centered oxidation to form a Ni(III) metallacyclobutane followed by facile ring fragmentation to release the olefin product. Intriguingly, a similar fragmentation is also induced by reaction with Brookhart's acid containing a non‐coordinating BAr^F^ anion. This suggests that oxidative protonation at nickel to generate a Ni(IV)–H species may lead to rapid [2+2] cycloreversion instead of the more expected C−H reductive elimination without a stabilizing counteranion.

These observations show the capacity of nickelacyclobutanes for the activation of element–hydrogen bonds, which may also constitute potential deactivation pathways for catalytic reactions relying on such intermediates. Furthermore, higher oxidation state nickelacyclobutanes were found to be highly reactive species prone to fragmentation and ring opening by Ni–C cleavage. Overall, the study of nickelacyclobutane reactivity provides a comprehensive experimental basis to support mechanistic proposals in catalytic reactions involving nickelacyclobutane intermediates and will guide the future development of such reactions.

## Supporting Information

Deposition numbers 2401206 (for **5**), 2401207 (for **7**), 2401208 (for **9**) contain the supplementary crystallographic data for this paper. These data are provided free if charge by the joint Cambridge Crystallographic Data Centre and Fachinformationszentrum Karlsruhe Access Structures service. https://www.ccdc.cam.ac.uk/structures

The authors have cited additional references within the Supporting Information.[^12^, ^17^, ^24^, ^47^, ^49^, ^50^, ^51^, ^52^, ^53^, ^54^, ^55^]

## Conflict of Interests

The authors declare no conflict of interest.

1

## Supporting information

As a service to our authors and readers, this journal provides supporting information supplied by the authors. Such materials are peer reviewed and may be re‐organized for online delivery, but are not copy‐edited or typeset. Technical support issues arising from supporting information (other than missing files) should be addressed to the authors.

Supporting Information

## Data Availability

The data that support the findings of this study are available in the supplementary material of this article.
