# Supplementary material for: Addition and Oxidation Reactivity of a Pentacoordinate Nickelacyclobutane
Source: Chemistry. 2025 Jan 8;31(14):e202404133. doi: 10.1002/chem.202404133 (PMC11886766; doi:10.1002/chem.202404133)
Supplement: Supplementary file 1 — Supporting Information [file CHEM-31-e202404133-s001.pdf]

Chemistry–A European Journal

Supporting Information

Addition and Oxidation Reactivity of a Pentacoordinate Nickelacyclobutane

María L. G. Sansores-Paredes, Martin Lutz, and Marc-Etienne Moret*

Supporting information

María L. G. Sansores-Paredes[1], Martin Lutz[2] and Marc-Etienne Moret[1]*

[1]Organic Chemistry & Catalysis, Institute for Sustainable and Circular Chemistry, Utrecht University, 3584 CG Utrecht, The Netherlands.
Email corresponding author: M.moret@uu.nl
[2]Structural Biochemistry, Bijvoet Centre for Biomolecular Research, Utrecht University, 3584 CG Utrecht, The Netherlands.

Table of contents

1. Experimental section S1

1.1 General information S1

1.2 Physical methods S1

1.3 Experimental section S1

2. Additional discussion S6

2.1 Reaction of nickelacyclobutane (1) with H_2 .... S6

2.2 Reaction of nickelacyclobutane (1) with $FeCp_2BF_4$ S8

2.3 Reaction of nickelacyclobutane (1) with Brookhart's acid and comparison with one-electron oxidation reaction S12

3. Spectra of new compounds S16

4. X-ray crystal structure determinations S29

5. DFT calculations S32

5.1 General information S32

5.2 Reactivity with H_2 .... S32

5.3 Reactivity with terminal alkynes S33

5.4 Methathesis after one electron oxidation of the nickelacyclobutane S33

5.5 Reactivity with Bronsted acids S36

5.4.1 HCl S36

5.4.2 Brookhart's acid S38

5.6 Table of energies S40

6. Literature references S42

1. Experimental section

1.1 General information

All reactants were purchased from commercial sources and used as received without further purification. Additionally, 1-ethynyl-4-fluorobenzene, HCl in Et_2O, and $FeCp_2BF_4$ were stored in the glovebox. All the reactions were performed under an N_2(g) atmosphere using standard Schlenk line or glovebox techniques. Deuterated solvents were purchased from Cambridge Isotope Laboratory Incorporation (Cambridge, USA), degassed by freeze pump procedure, and stored over molecular sieves before use. Common solvents were dried using a MBRAUN MB SPS-80 purification system and THF was purified by distillation from a THF/sodium/benzophenone suspension. Brookhart's acid was synthetized according to the literature procedure and stored in the glovebox.[1] Nickelacyclobutane (**1**) was synthetized according to the procedure described in the literature.[2] The bulk purity of the reported compounds was determined by NMR analysis.

1.2 Physical methods

^{1}H, ^{13}C, ^{11}B, ^{19}F, and ^{31}P NMR spectra (400, 101, 128, 376, and 161 MHz respectively) were recorded on an Agilent MR400 or a Varian AS400 spectrometer at 25 °C unless it is stated differently. ^{1}H and ^{13}C NMR chemical shifts relative to tetramethylsilane were referenced to the residual solvent resonance. ^{11}B NMR chemical shifts were referenced to $BF_3{\cdot}OEt_2$, ^{19}F NMR chemical shifts were referenced to $CFCl_3$ and ^{31}P NMR chemical shifts were referenced to 85% aqueous H_3PO_4 solution; all externally. Infrared spectra were recorded using a Perkin Elmer Spectrum One FT-IR spectrometer under N_2 flow. EPR spectra were recorded on a Bruker EMX PLUS 6000 Gauss machine with ER 041 XG X-band Microwave Bridge.

1.3 Experimental section

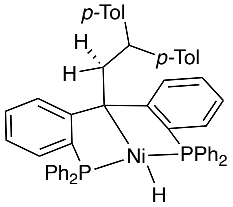

[PC(CH_2CH(*p*-Tol)$_2$)P]NiH (2): Nickelacyclobutane **1** (10 mg, 0.0125 mmol) was dissolved in d^8-toluene (0.8 mL) and the solution was placed in a J-Young NMR tube. The sample was degassed by two freeze-pump-thaw cycles and, while the tube was immersed in liquid nitrogen, hydrogen gas was introduced. The solution was warmed up to room temperature and analyzed. An equilibrium between hydride **2** and (^{Ph}bppe^{H,CHptol2})Ni(H_2) **2'** was identified by its NMR spectroscopical properties matching a previous report in the literature.[3] Further

details on this equilibrium mixture, which can also be accessed starting with the olefin complex **3,** were further investigated in a previous report.[3]

Equilibrium mixture at –40 °C: ^{1}H NMR (400 MHz, d^{8}-toluene, –40 °C): δ(ppm) 7.84–7.74 (m, 2H), 7.68 (d, *J*= 8.1 Hz, 2H), 7.54 (s, 4H), 7.45 (d, *J*= 7.7 Hz, 1H), 7.38–7.18 (m, 11H), 6.95–6.85 (m, 12H), 6.84–6.73 (m, 11H), 4.76 (s, CH_{olefin} complex 3), 4.40 (s, CH_{olefin} complex (^{Ph}bppe^{H,CHptol2})Ni(H_2)), 3.80 (t, $^{3}J_{H,H}$= 8.4 Hz, 1H, C*H*), 3.12 (b, 2H, CH_2), -2.09 (b, Ni-H_2), -14.29 (t, $^{2}J_{H,P}$= 64.3 Hz, 1H, Ni-*H*).

^{31}P{H^{1}} NMR (162 MHz, d^{8}-tol, –40 °C): δ(ppm) 40.6 (s, 2P), 28.2 (d, $^{2}J_{P,P}$= 53.3 Hz, 1P), 32.8 (d, $^{2}J_{P,P}$= 60.2 Hz, 1P), 15.5 (d, $^{2}J_{P,P}$= 58.7 Hz, 1P), 11.3 (d, $^{2}J_{P,P}$= 53.6 Hz, 1P).

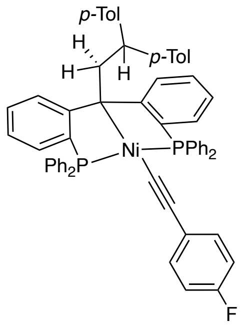

[PC(CH$_2$CH(*p*-Tol)$_2$)P]Ni(CC(C$_6$H$_5$F) (4): 1.2 equiv. of 1-ethynyl-4-fluorobenzene (0.015 mmol, 1.7 μL) were added to a solution of complex **10** (10 mg, 0.0125 mmol) in approximately 0.6 mL of C_6D_6. The solution was immediately transferred to a J-Young NMR tube and NMR data was recorded to corroborate full conversion. The compound was identified by its spectroscopical properties matching a previous report in the literature.[4]

^{1}H NMR (400 MHz, C_6D_6, 25 °C): δ(ppm) 7.89 (d, *J*= 5.7 Hz, 4H, Ar-*H*), 7.53 (d, *J*= 8.1 Hz, 2H, Ar-*H*), 7.45–7.36 (m, 4H, Ar-*H*), 7.33–7.25 (m, 2H, Ar-*H*), 7.22–7.17 (m, 6H, Ar-*H*), 7.12 (d, *J*= 7.8 Hz, 2H, Ar-*H*), 7.01 (d, *J*= 8.0 Hz, 4H, Ar-*H*), 6.93 (t, *J*= 6.6 Hz, 6H, Ar-*H*), 6.87 (t, *J*= 7.2 Hz, 8H, Ar-*H*), 6.66 (t, *J*= 8.8 Hz, 2H, Ar-*H*), 4.01 (t, $J_{H,H}$= 7.1 Hz, 1H, C*H*), 2.99 (d, $J_{H,H}$= 7.7 Hz, 2H, CH_2), 2.14 (s, 6H, CH_3).

^{31}P{^{1}H} NMR (162 MHz, C_6D_6, 25 °C): δ(ppm) 35.2 (s, 2P). ^{19}F NMR (376 MHz, C_6D_6, 25 °C): δ(ppm) -115.76 – -119.51 (m, 1F).

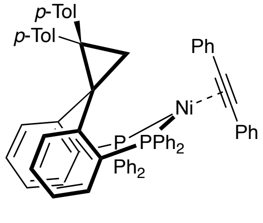

[P(cyclopropane)P]Ni(DPA) (5): Nickelacyclobutane **1** (20 mg, 0.025 mmol) was dissolved in benzene (1 mL). 1.2 equiv. of diphenylacetylene (0.03 mmol, 5.3 mg) were dissolved in benzene (1 mL) and added to the nickelacyclobutane solution. The solution was stirred for 24 h, the solvent was evaporated until approximately a third of the original volume and

hexane (2 mL) was added, prompting precipitation of the product. The solid was filtered and washed with hexane (2 X 0.5 mL) and dried in vacuum to obtain a yellow powder (21 mg, 86%). Crystals suitable for XRD were obtained by slow vapor diffusion of hexane into a concentrated toluene solution.

^{1}H NMR (400 MHz, C_6D_6, 25 °C): δ(ppm) 7.79 (s, 4H, Ar-*H*), 7.64–7.58 (m, 2H, Ar-*H*),), 7.06 (d, *J*= 8.0 Hz, 6H, Ar-*H*), 6.99–6.86 (m, 21H, Ar-*H*), 6.83 (t, *J*= 7.6 Hz, 2H, Ar-*H*), 6.77 (d, *J*= 8.1 Hz, 4H, Ar-*H*), 6.63 (d, *J*= 7.9 Hz, 4H, Ar-*H*), 6.53 (t, *J*= 7.3 Hz, 2H, Ar-*H*), 3.74 (s, 2H, C*H*$_2$), 1.99 (s, 6H, C*H*$_3$).

^{31}P{^{1}H} NMR (162 MHz, C_6D_6, 25 °C): δ(ppm) 31.8 (s, 2P).

^{13}C{^{1}H} NMR (101 MHz, C_6D_6, 25 °C): 148.0 (t, *J*= 7.2 Hz, Ar), 139.2 (s, Ar), 138.6 (s, Ar), 138.3 (s, Ar), 138.7 (d, *J*= 3.4 Hz, Ar or C_{alkyne}), 137.8 (t, *J*= 7.0 Hz, Ar), 136.8 (s, Ar), 136.6 (s, Ar), 136.3 (t, *J*= 3.4 Hz, Ar, or C_{alkyne}), 135.9 (s, Ar), 135.7 (d, *J*= 3.2, Ar), 135.5 (s, Ar), 134.7 (t, *J*= 7.1 Hz, Ar), 134.5 (s, Ar), 133.6 (t, *J*= 6.4 Hz, Ar), 132.1 (d, *J*= 34.0 Hz, Ar), 130.7 (s, Ar), 129.0 (s, Ar), 128.6 (s, Ar), 128.4 (s, Ar), 127.4 (s, Ar), 125.6 (s, Ar), 124.1 (s, Ar), 45.7 (t, *J*= 9.7 Hz, $C_{quaternary}$), 39.6 (t, *J*= 4.8 Hz, $C_{quaternary}$), 34.0 (s, *C*H$_2$), 20.9 (s, *C*H$_3$).
IR (cm^{-1}): 3053, 2957, 2923, 2853, 1796, 1671, 1588, 1512, 1434, 1260, 1197, 1091, 1026, 892, 810, 756, 744, 692, 535, 523, 483. The high sensitivity of this compound did not allow to obtain elemental analysis data.

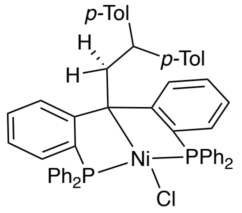

[PC(CH$_2$CH(*p*-Tol)$_2$)P]NiCl (7): Nickelacyclobutane **1** (20 mg, 0.025 mmol) was dissolved in 2 mL of toluene. A solution of HCl in ether (1M, 28 μL, 0.028 mmol) was added dropwise. The solution was stirred for 20 min, after which the solution was concentrated to a quarter of the original volume in vacuum. Hexane (1.5 mL) was added and the mixture was stirred. The resulting red precipitate was isolated by filtration, washed with hexane (0.5 mL), and dried in vacuum to yield a red powder (18 mg, 87%). Crystals suitable for XRD were obtained by slow vapor diffusion of hexane into a concentrated toluene solution of **7**.

^{1}H NMR (400 MHz, C_6D_6, 25 °C): δ(ppm) 8.04–7.89 (m, 4H, Ar-*H*), 7.52 (q, *J*= 6.1 Hz, 4H, Ar-*H*), 7.28 (d, *J*= 8.1 Hz, 2H, Ar-*H*), 7.20 (m, *J*= 3.8 Hz, 2H, Ar-*H*), 6.91 (m, *J* = 30.0, 15.3, 7.7 Hz, 13H, Ar-*H*), 6.79 (t, *J* = 7.3 Hz, 2H, Ar-*H*), 5.15 (t, $J_{H\text{-}H}$ = 6.6 Hz, 1H, -CH$_2$-C*H*), 2.77 (d, $J_{H\text{-}H}$ = 6.8 Hz, 2H, C*H*$_2$-CH), 2.10 (s, 6H, C*H*$_3$). Some aromatic signals are obscured by the residue solvent peak.

^{31}P{^{1}H} NMR (162 MHz, C_6D_6, 25 °C): δ(ppm) 24.9 (s, 2P).

^{13}C{^{1}H} NMR (101 MHz, C_6D_6, 25 °C): δ(ppm) 162.2 (t, *J*= 18.9 Hz, Ar), 143.9 (s, Ar), 138.1–137.1 (m, Ar), 135.0 (s, Ar), 134.8 (s, Ar), 134.6 (d, *J*= 6.5 Hz, Ar), 134.4 (s, Ar), 133.5 (t, *J*= 5.9 Hz, Ar), 130.9 (t, *J*= 20.2 Hz, Ar), 130.2 (d, *J*= 26.6 Hz, Ar), 129.6 (s, Ar), 129.2 (s, Ar),

128.7 (t, J= 4.9 Hz, Ar), 127.4 (t, J= 8.7 Hz, Ar), 126.69 (s, Ar), 60.4 (t, J= 5 Hz, C-CH_2), 57.6 (s, C-CH_2), 51.67 (s, CH_2-CH), 21.1 (s, CH_3).
IR (cm^{-1}): 3051, 2961, 2923, 2853, 1509, 1434, 1260, 1097, 1066, 1022, 909, 801, 743, 718, 692, 570, 517. The high sensitivity of this compound did not allow to obtain elemental analysis data.

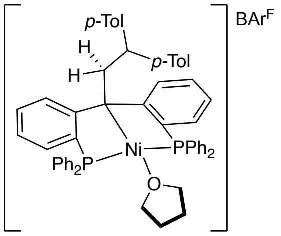

{[PC(CH_2CH(*p*-Tol)$_2$)P]Ni(THF)}BAr^F (9): Compound [(^{Ph}bppe^{H,CHptol2})Ni]$_2$$N_2$ (**3,** 20 mg, 0.012 mmol) was dissolved in THF (3 mL). A solution of Brookhart's acid (25 mg, 0.025 mmol) in THF (2 mL) was added dropwise. The solution was stirred for 1 h, after which the solution was concentrated down to a quarter of the original volume in vacuum. Hexane (2 mL) was added, and a pink precipitate was observed. The solid was isolated by filtration, washed with hexane (0.5 mL) and dried in vacuum to obtain a pink powder (35 mg, 91%). ***Alternative synthesis:*** [PCH_2CH(*p*-Tol)$_2$P]NiCl (20 mg, 0.024 mmol) was dissolved THF (2 mL). NaBAr^F (22 mg, 0.024 mmol) suspended in 1 mL of THF was added dropwise, and the reaction mixture was stirred for 1 h. The solution was filtered, and the solvent was removed in vacuum. The pink mixture was redissolved in toluene (0.5 mL) and hexane (3 mL) was added. The precipitate was isolated by filtration and washed with hexane (1 mL). The solid was dried under vacuum to obtain the product as a pink powder (30 mg, 78%). Crystals suitable for X-ray diffraction were obtained by slow vapor diffusion of hexane into a concentrated toluene solution.
^{1}H NMR (400 MHz, C_6D_6, 25 °C): δ(ppm) 8.41–8.35 (m, 8H, Ar-H BAr^F fragment), 7.64 (s, 4H, Ar-H BAr^F fragment), 7.42 (q, J = 5.9 Hz, 4H, Ar-H), 7.21 (d, J= 7.0 Hz, 2H, Ar-H), 7.06–6.90 (m, 8H, Ar-H), 6.86 (t, J= 7.6 Hz, 4H, Ar-H), 6.79 (d, J= 9.1 Hz, 13H, Ar-H), 4.80 (t, J_{H-H}= 5.2 Hz, 1H, CH_2-CH), 2.81 (b, 4H, THF), 2.19 (d, J_{H-H}= 5.8 Hz, 2H, CH_2-CH), 2.05 (s, 6H, CH_3), 1. (b, 4H, THF).
^{31}P{^{1}H} NMR (162 MHz, C_6D_6, 25 °C): δ(ppm) 23.2 (s, 2P).
^{13}C{^{1}H} NMR (101 MHz, C_6D_6, 25 °C): δ(ppm) 162.8 (dd, J = 99.6, 50.0 Hz, Ar), 159.5 (t, J= 17.5 Hz, Ar), 143.3 (s, Ar), 136.4 (s, Ar), 135.4 (s, Ar), 133.9 (s, Ar), 133.5 (t, J= 6.7 Hz, Ar), 132.3 (s, Ar), 132.1 (t, J= 5.9 Hz, Ar), 131.7 (s, Ar), 131.0 (s, Ar), 129.7 (t, J= 5.4 Hz, Ar), 129.4 (t, J= 4.8 Hz, Ar), 128.5–128.3 (m, Ar), 127.7 (s, Ar), 127.0 (t, J= 8.1 Hz, Ar), 126.6 (s, Ar), 123.9 (s, Ar), 121.2 (s, Ar), 118.1 (t, J= 5 Hz, Ar), 56.6 (t, J= 7Hz, C-CH_2), 56.2 (t, J=4, Hz, C-CH_2), 52.0 (s, CH_2-CH), 20.7 (s, CH_3). ^{11}B (128 MHz, THF-d^8, 25 °C): δ(ppm) -5.97 (s).
^{19}F NMR (376 MHz, THF-d^8, 25 °C): δ(ppm) -62.2 (s).

IR (cm^{-1}): 3059, 2962, 2928, 1609, 1510, 1482, 1459, 1436, 1354, 1276, 1124, 886, 839, 744, 713, 694, 682, 670, 670, 601. The high sensitivity of this compound did not allow to obtain elemental analysis data.

Reaction of nickelacyclobutane 1 with $FeCp_2BF_4$: Nickelacyclobutane **1** (10 mg, 0.0125 mmol) were dissolved in THF (1 mL). A suspension of $FeCp_2BF_4$ (3.5 mg, 0.0125 mmol) in THF (1 mL) was added dropwise, causing an immediate color change to dark green. The solution was stirred for 10 min and concentrated down to 1 mL in vacuum. Hexane (3 mL) was added, resulting in the formation of a dark green precipitate. The solid was isolated by filtration, redissolved in THF and reprecipitated by adding hexane. The product was then washed with hexane (1 mL) and dried under vacuum to obtain 4 mg of a green powder. The obtained solid was NMR silent. For EPR measurement, the powder obtained was dissolved in toluene. The high sensitivity of this compound did not allow to obtain further spectroscopic data. For the detection of 1,1-di(*p*-tolyl)ethylene, the reaction was repeated using d^8-THF without isolation of the green powder. After stirring for 10 min, approximately half of the reaction mixture was transferred to a J Young NMR tube and spectra were recorded.

Reaction with Brookhart's acid: Nickelacyclobutane **1** (20 mg, 0.025 mmol) was dissolved in THF (2 mL). A solution of Brookhart's acid (25.3 mg, 0.025 mmol) in THF (3 mL) was added dropwise. The solution was stirred for 10 min, after which it was concentrated down to 0.5 mL, and 2 mL of hexane were added. A pink oil precipitated out and the solvent was removed by decantation.

^{1}H NMR (400 MHz, THF-d^8, 25 °C): δ(ppm) 14.99, 10.61, 7.67, 1.73.

^{11}B NMR (128 MHz, THF-d^8, 25 °C): δ(ppm) -4.69 (s).

^{19}F NMR (376 MHz, THF-d^8, 25 °C): δ(ppm) -63.4 (s).

The high sensitivity of this compound did not allow to obtain IR and elemental analysis data. For the detection of 1,1-di(*p*-tolyl)ethylene, the reaction was repeated using d^8-THF without isolation of the oil. After stirring for 10 min, approximately half of the reaction mixture was transferred to a J Young NMR tube and spectra were recorded.

2. Additional discussion

2.1 Reaction of nickelacyclobutane (1) with H_2

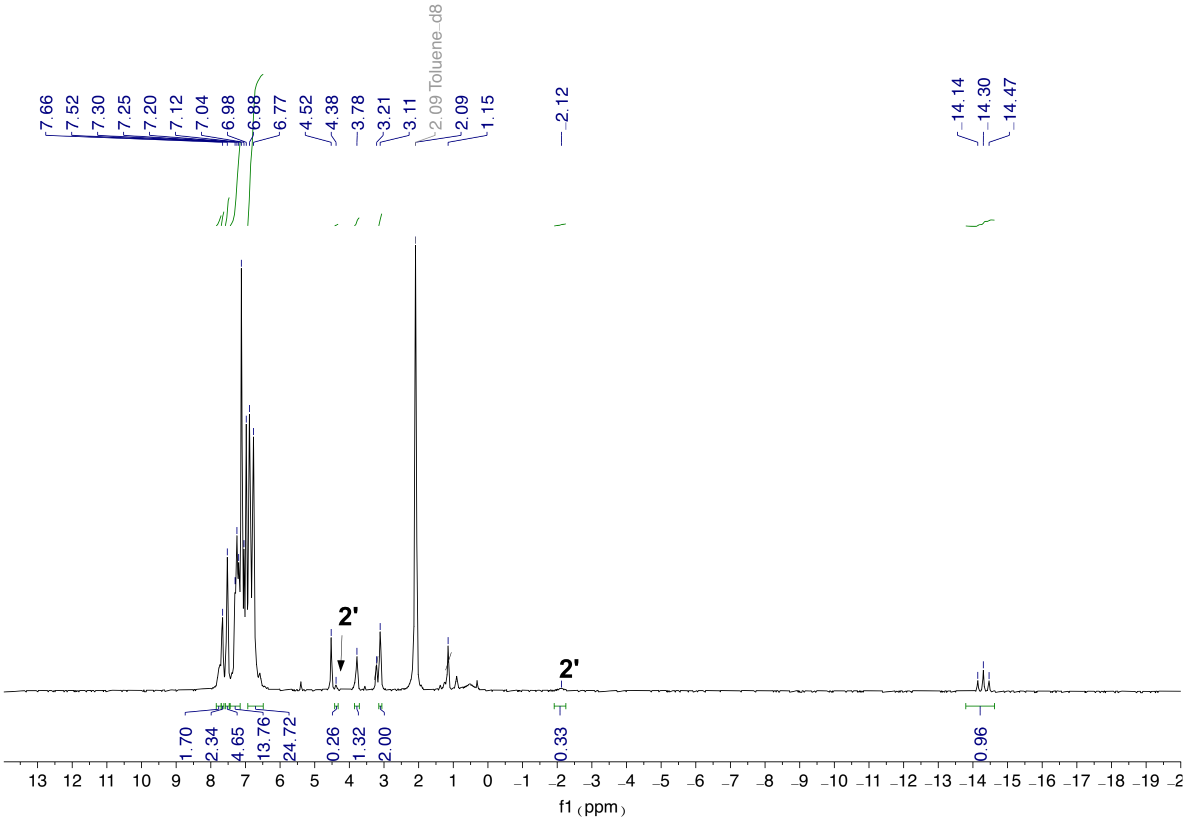

Figure S1. ^1H NMR at –40 °C in d^8-toluene of the reaction of nickelacyclobutane (**1**) with H_2. Characteristic peaks of complex **2’** are identified.[3]

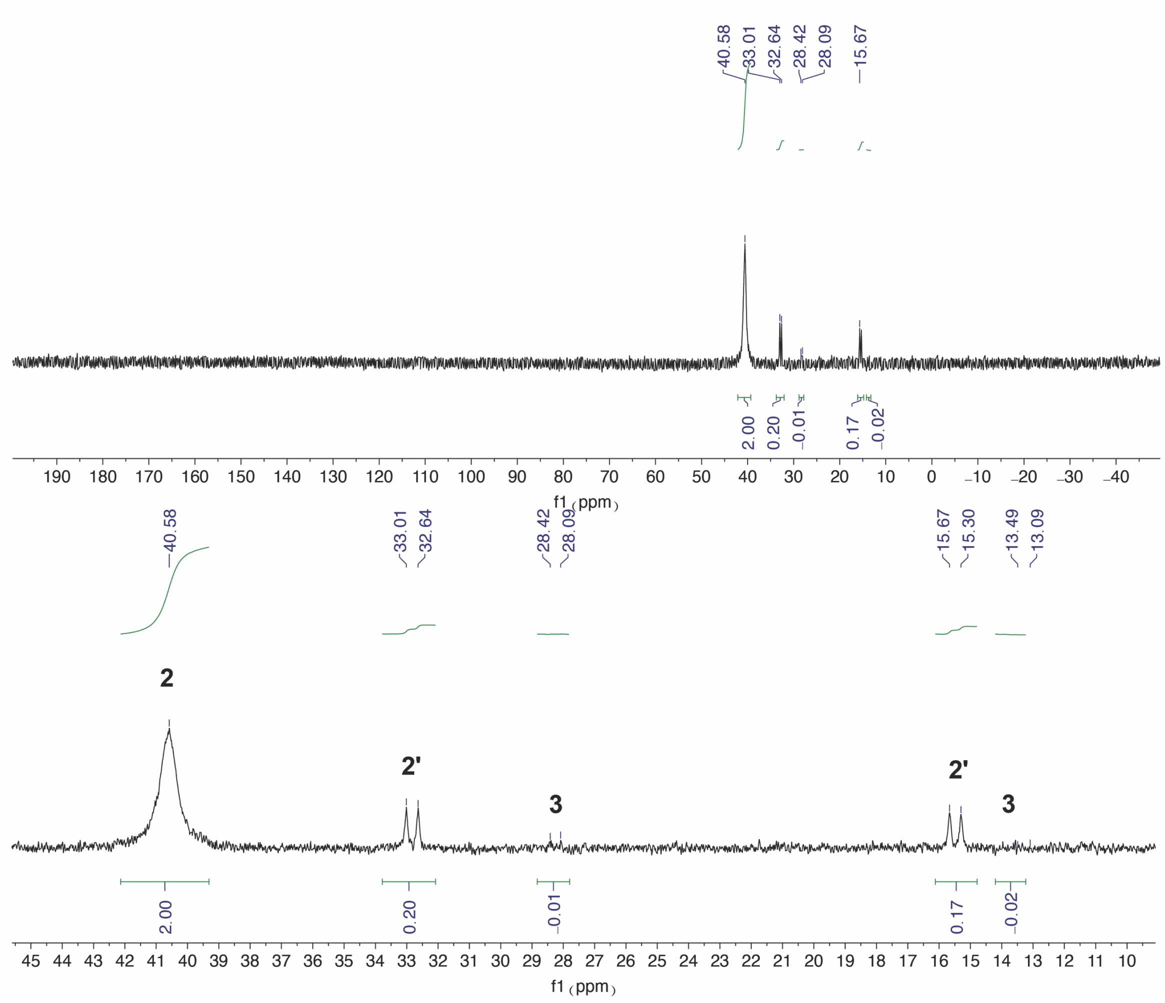

Figure S2. $^{31}P\{^{1}H\}$ NMR at –40 °C in d^8-toluene of the reaction of nickelacyclobutane (**1**) with H_2. Characteristic peaks of complex **2'** and **3** are identified.[3]

2.2 Reaction of nickelacyclobutane (1) with $FeCp_2BF_4$

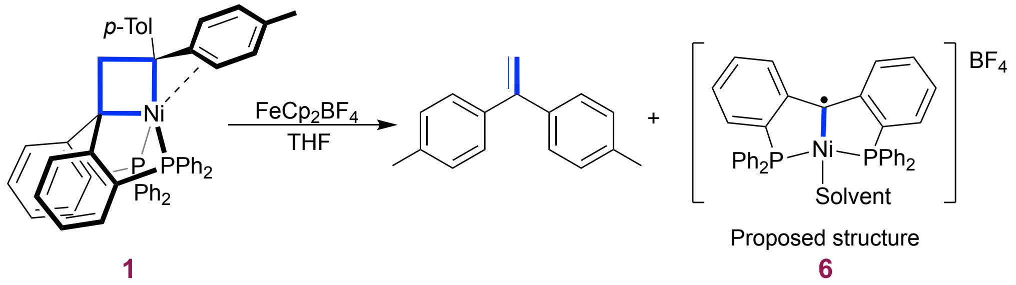

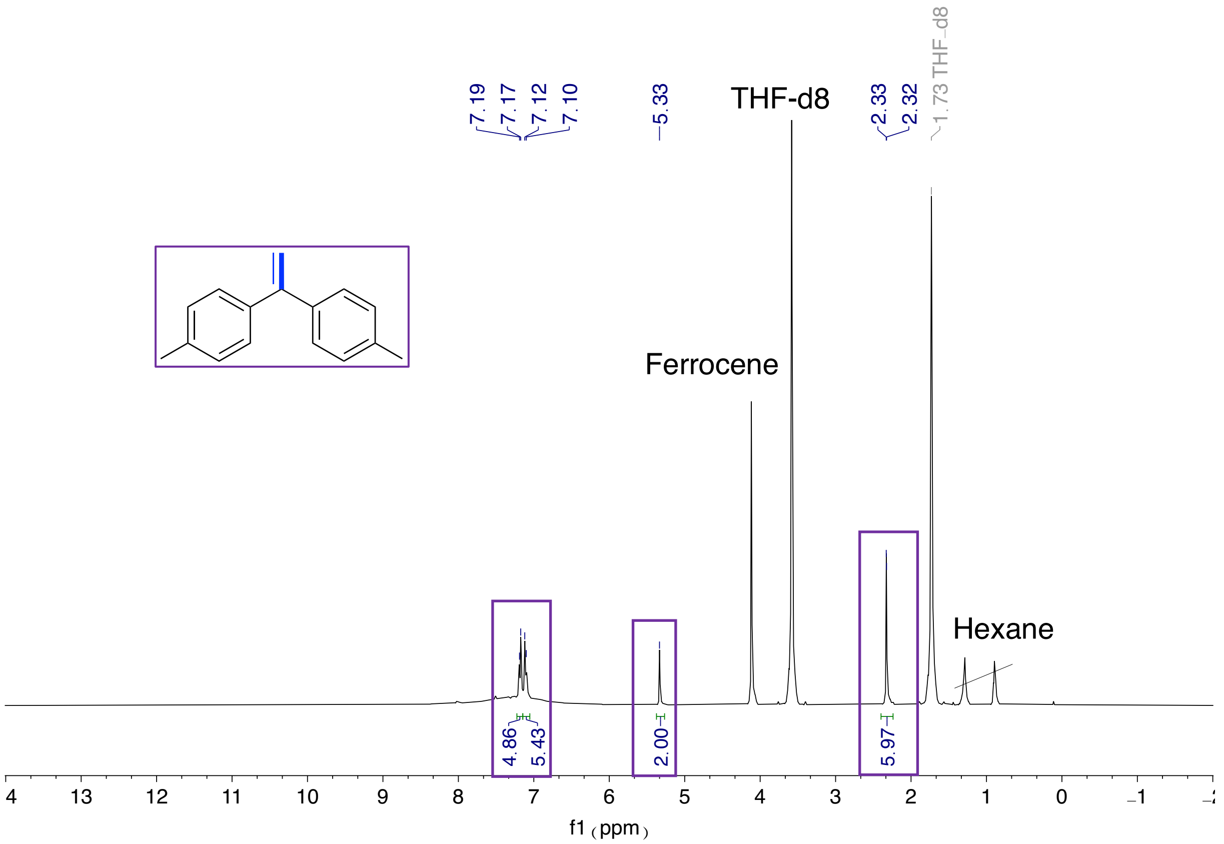

Figure S3. ^{1}H NMR in d[8]-THF of the crude reaction of nickelacyclobutane (**1**) with $FeCp_2BF_4$ at 25 °C.

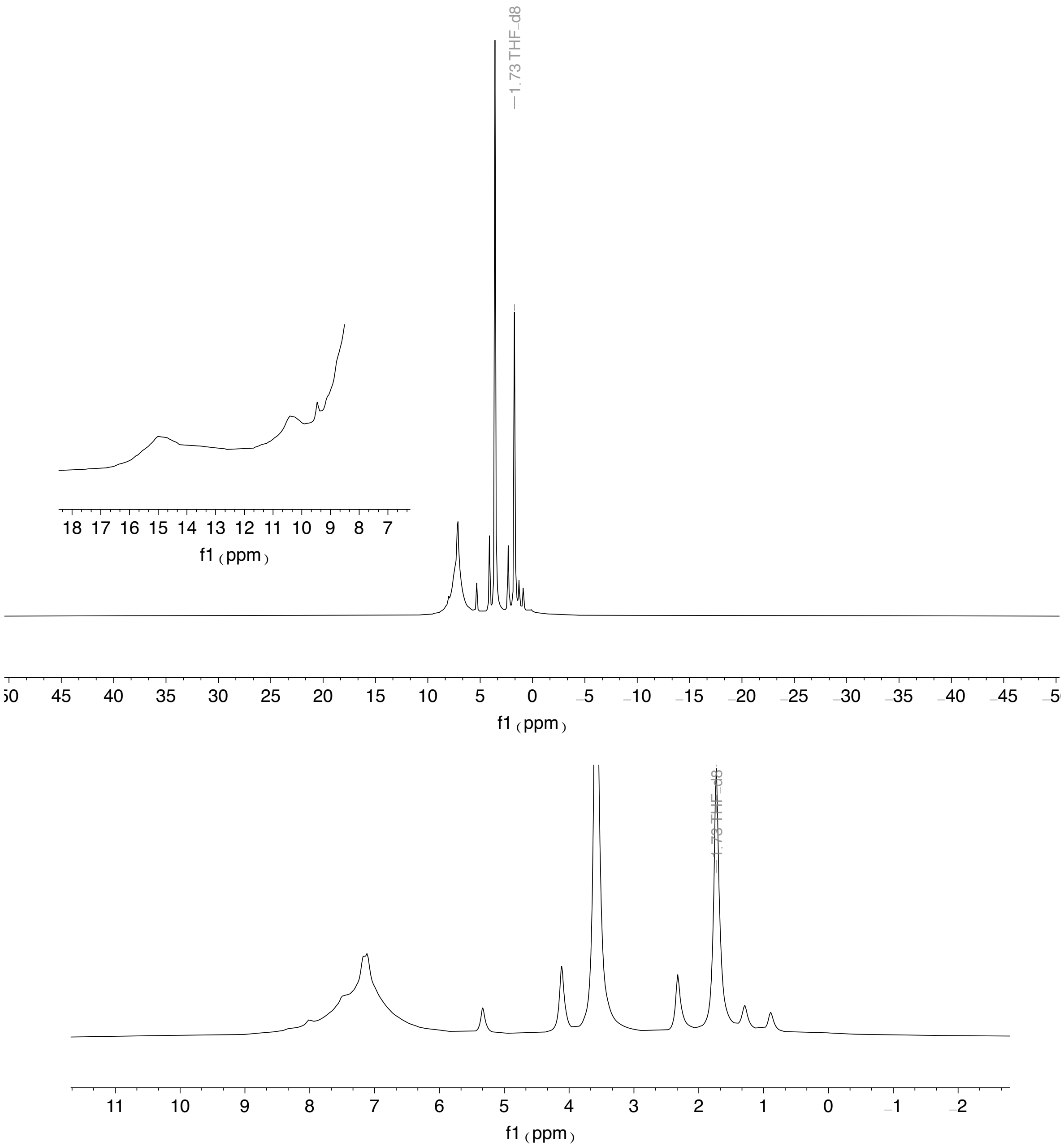

Figure S4. ^{1}H NMR in d^8-THF of the crude reaction of nickelacyclobutane **1** with $FeCp_2BF_4$ with paramagnetic settings at 25 ºC.

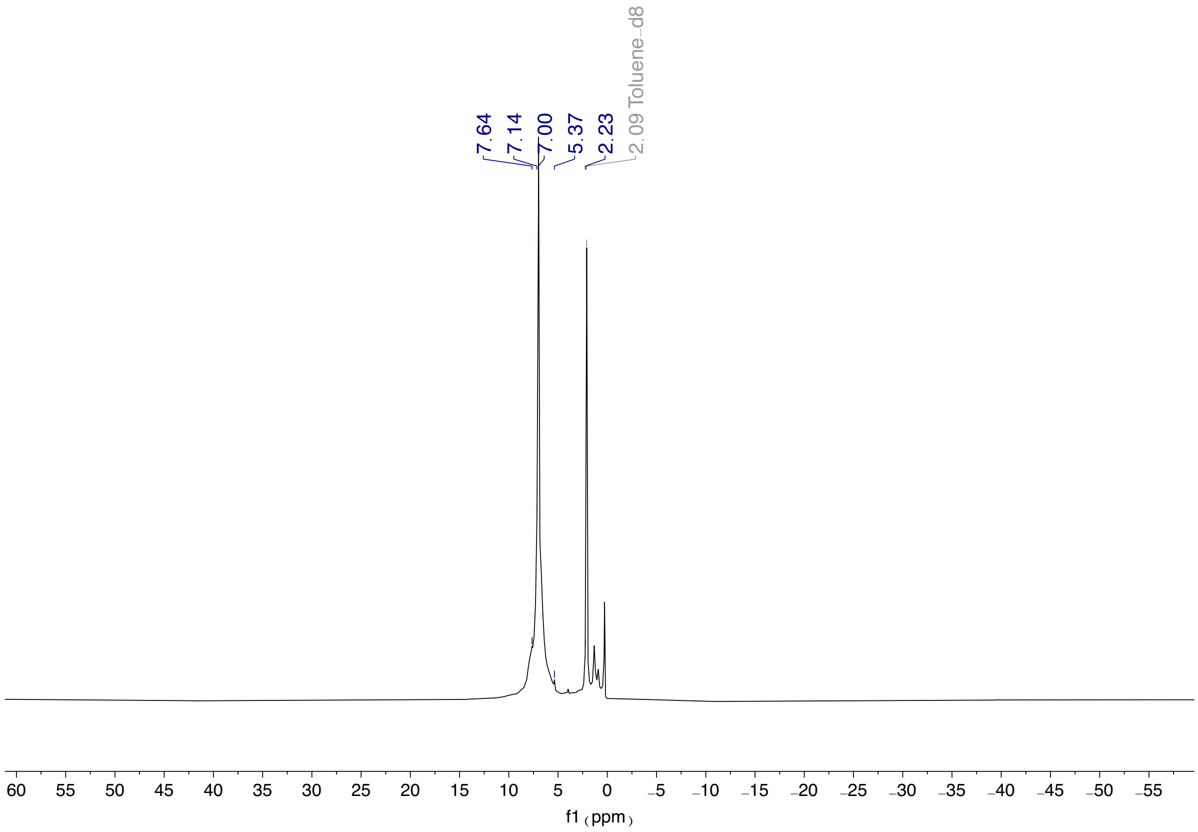

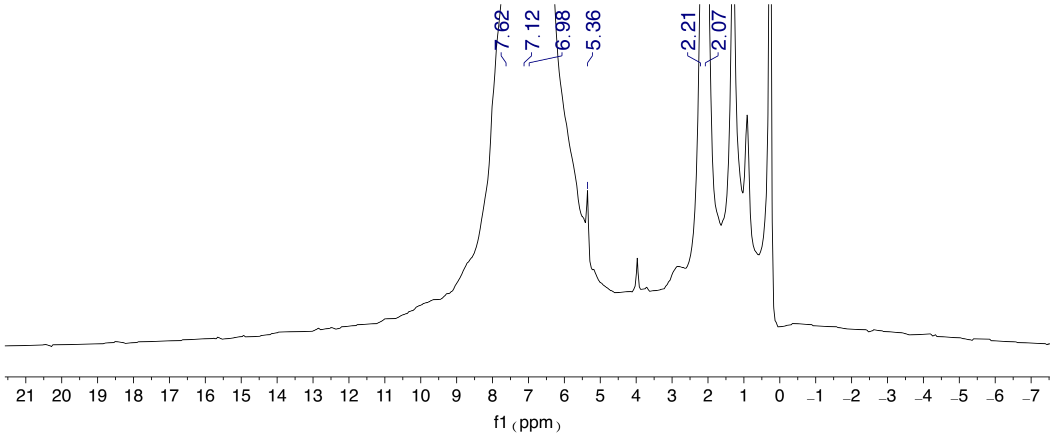

Figure S5. ^{1}H NMR in d^8-toluene of reaction of nickelacyclobutane (**1**) with $FeCp_2BF_4$ after workup with paramagnetic settings at 25 ºC.

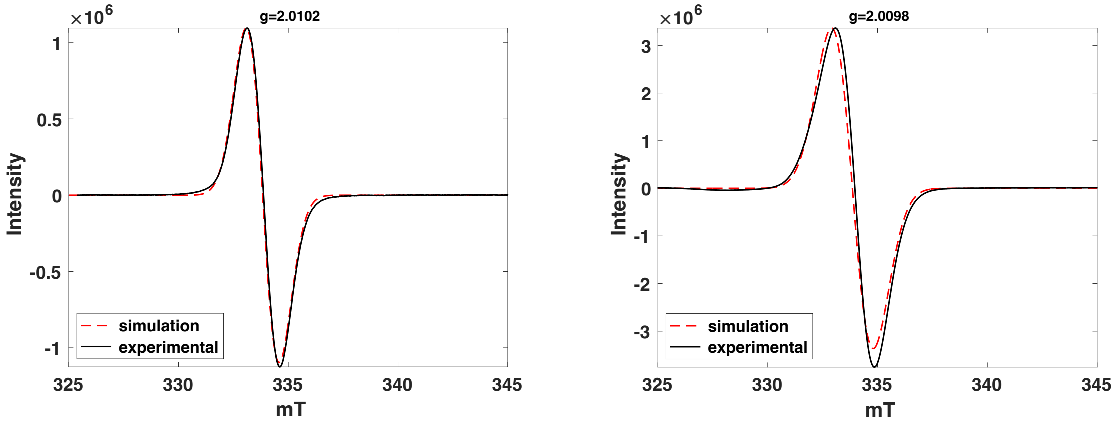

g= 2.0102 (298 K) g= 2.0098 (90 K)

Figure S6. EPR spectra recorded at different temperatures of the reaction of nickelacyclobutane (**1**) and $FeCp_2BF_4$ after workup in toluene. Proposed complex **6** peak is shown in black and simulated spectra are presented in red.

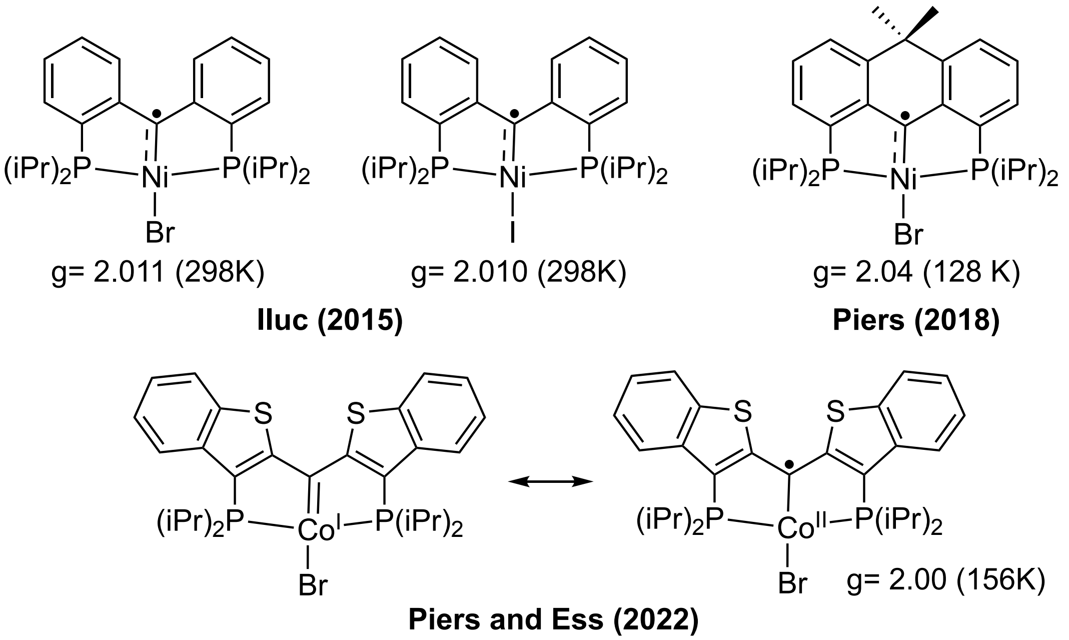

Figure S7. Examples of radical $PC_{carbene}P$ complexes reported in recent literature.[5-7]

2.3 Reaction of nickelacyclobutane (1) with Brookhart's acid and comparison with one-electron oxidation reaction

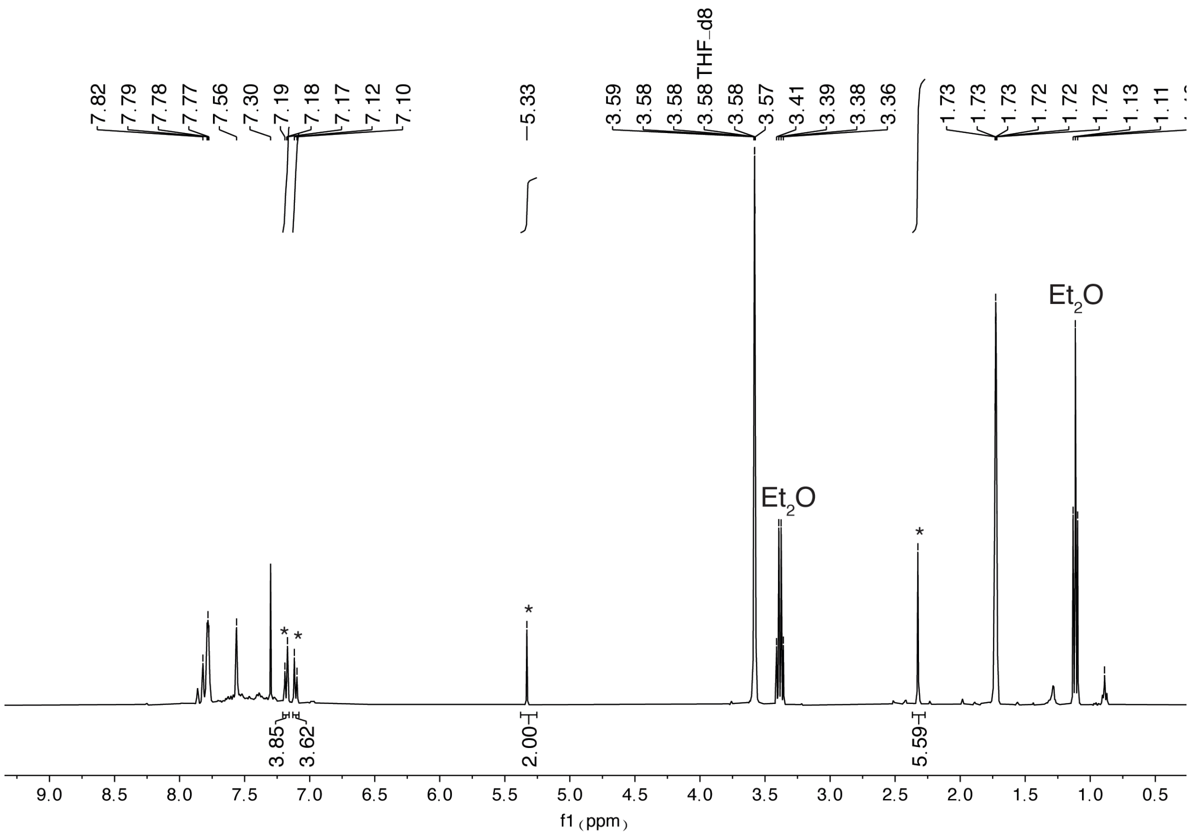

Figure S8. ^{1}H NMR of the crude reaction of nickelacyclobutane (**1**) with Brookhart's acid after 10 min in d^8-THF. Marked peaks (*) correspond to 1,1-di(*p*-tolyl)ethylene at 25 °C.

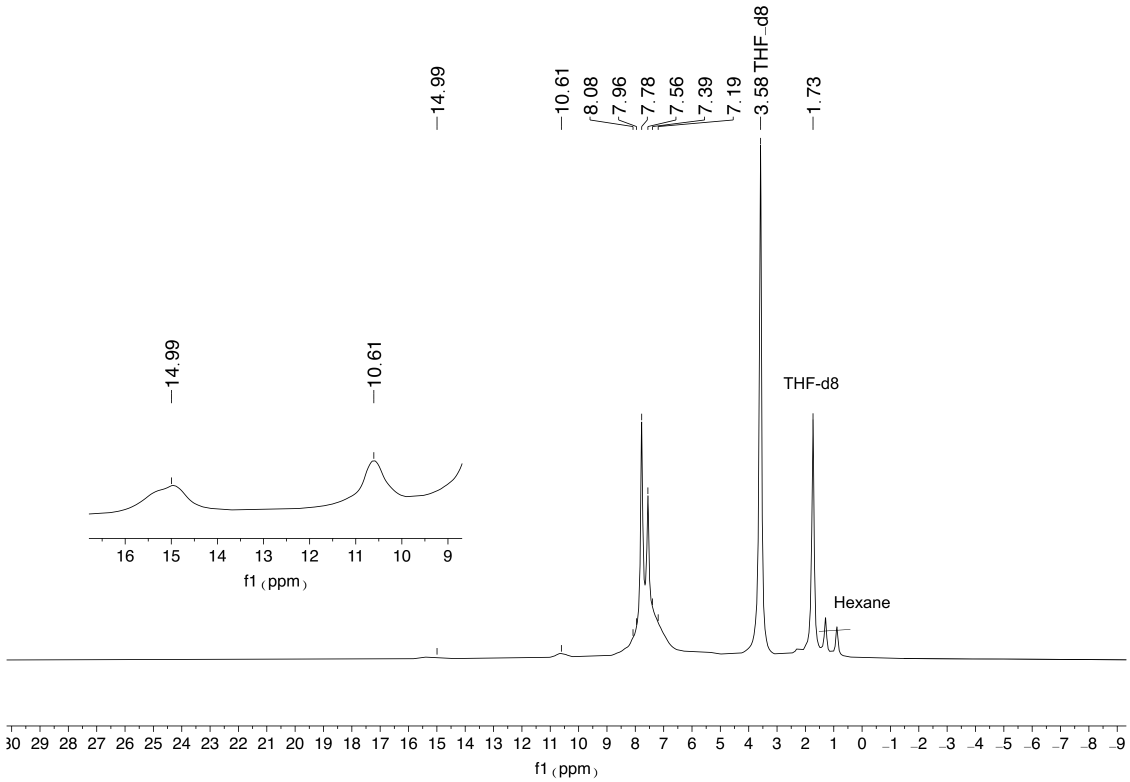

Figure S9. ^{1}H NMR in d^8-THF of the isolated oil from the reaction of nickelacyclobutane (**1**) with Brookhart's acid with paramagnetic settings at 25 °C.

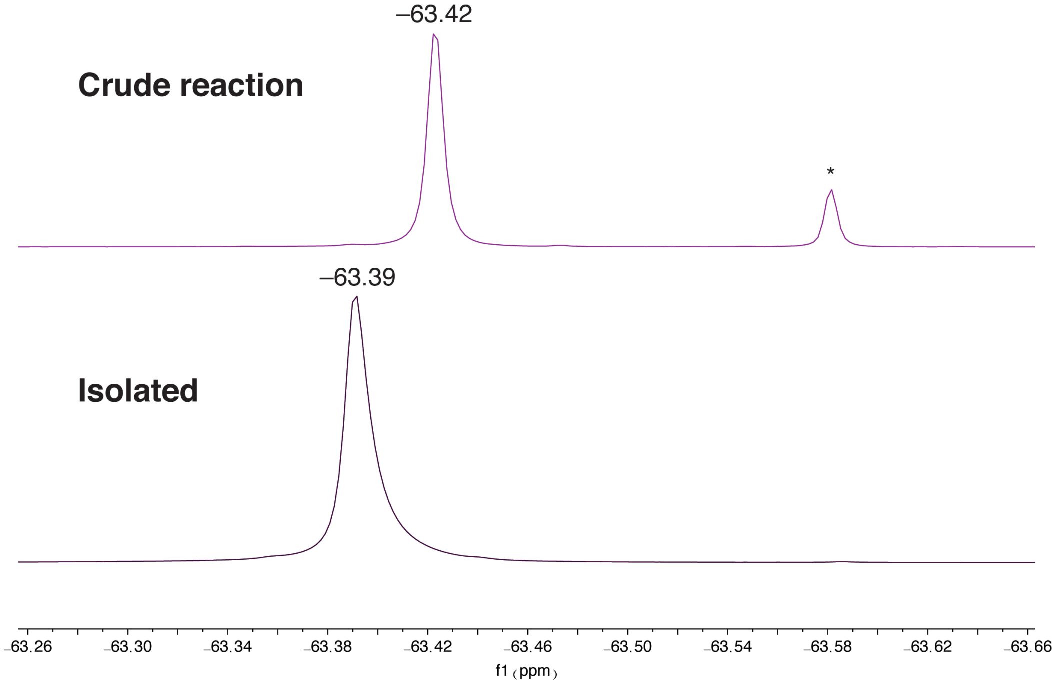

Figure S10. ^{19}F NMR comparison of the reaction of nickelacyclobutane (**1**) with Brookhart's acid. On top: crude reaction after 10 min. Marked peak correspond to Brookhart's acid. Down: isolated oil.

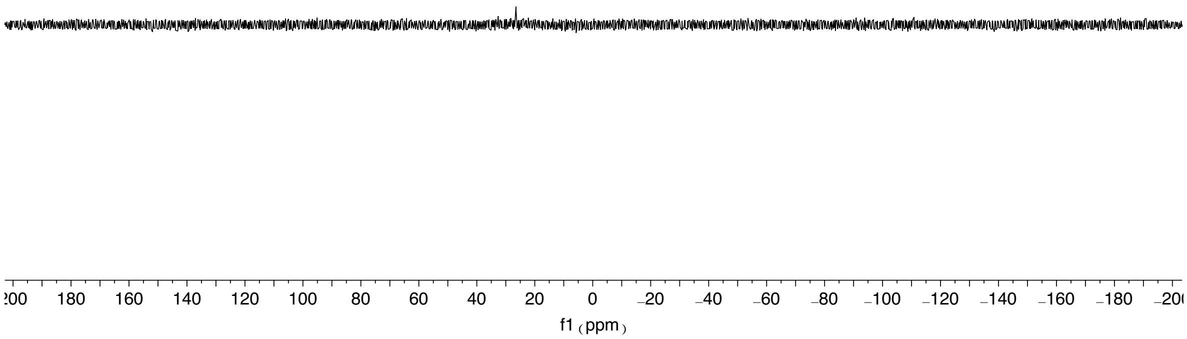

Figure S11. $^{31}P\{^{1}H\}$ NMR in d^8-THF of the isolated oil from the reaction of nickelacyclobutane (**1**) with Brookhart's acid with paramagnetic settings at 25 ºC.

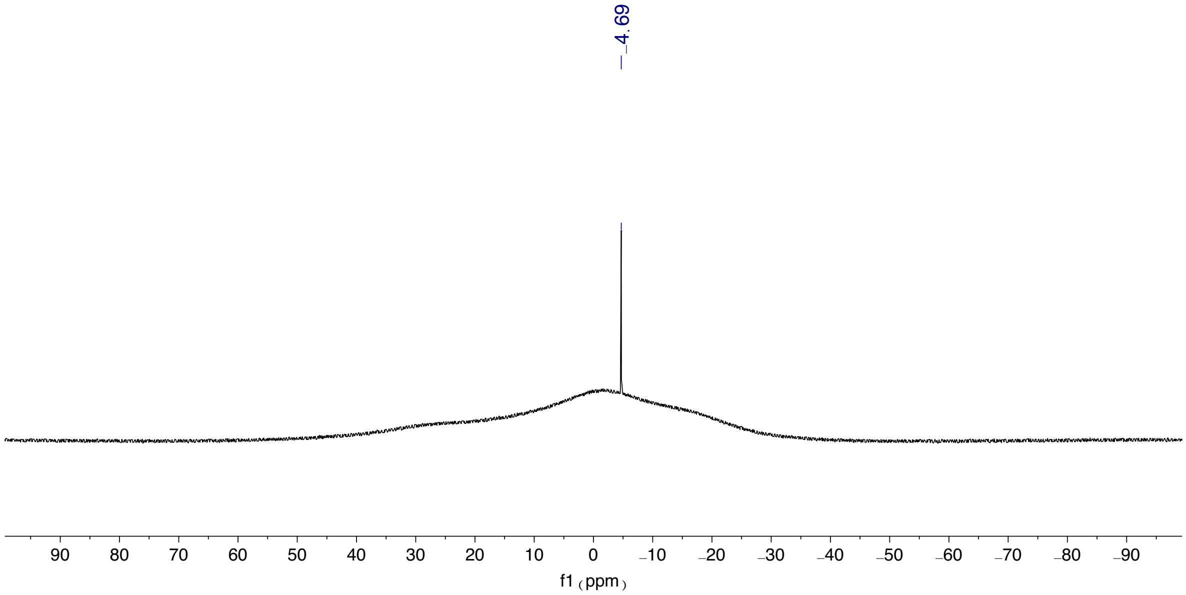

Figure S12. ^{11}B NMR in d^8-THF of the isolated oil from the reaction of nickelacyclobutane (**1**) with Brookhart's acid with paramagnetic settings at 25 ºC.

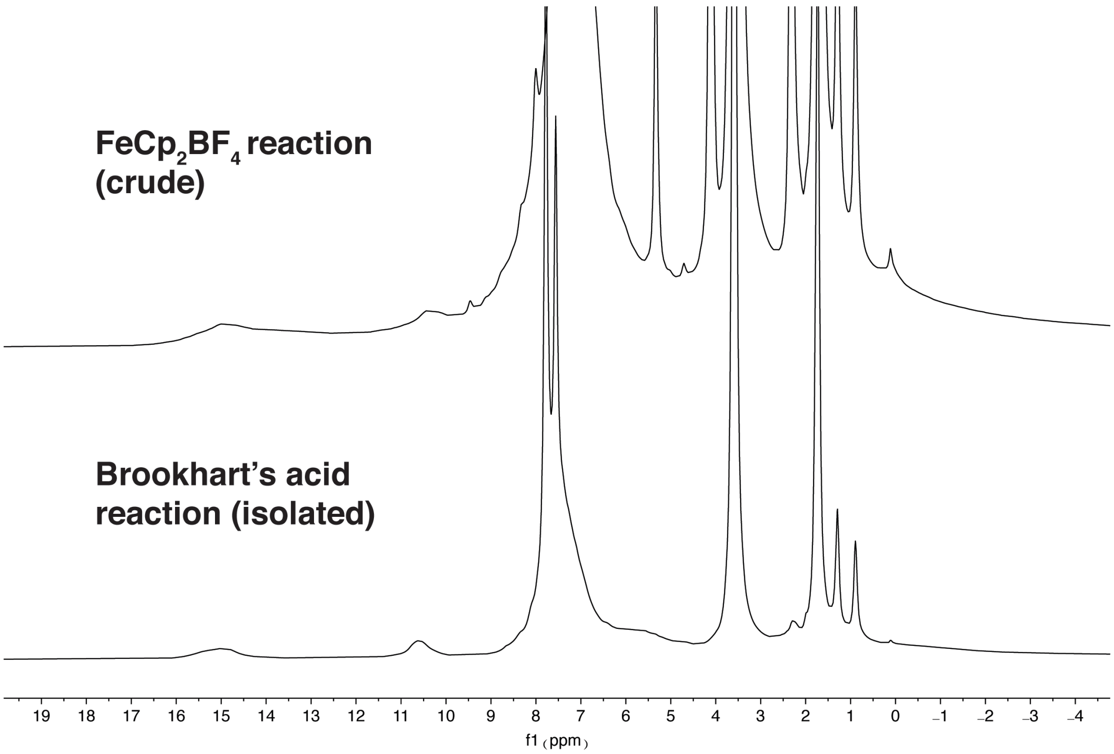

Figure S13. Comparison of ^1H NMR in d^8-THF of the crude reaction of nickelacyclobutane (**1**) with $FeCp_2BF_4$ and the isolated oil from the reaction of nickelacyclobutane (**1**) and Brookhart's acid with paramagnetic settings.

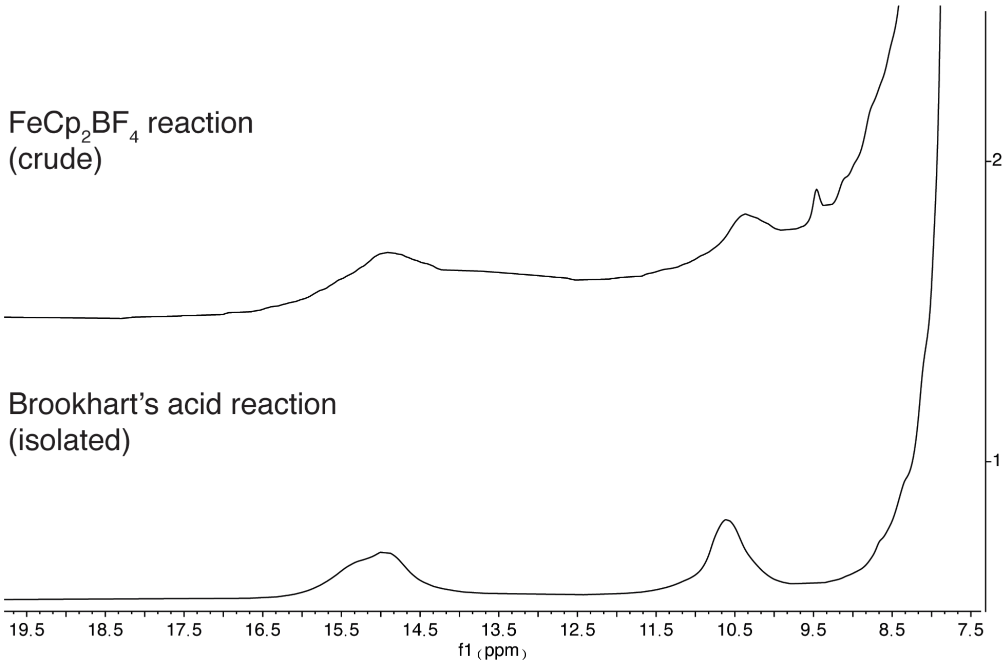

Figure S14. Comparison of ^1H NMR in d^8-THF (zoom 7.5-20 ppm) of the crude reaction of nickelacyclobutane (**1**) with $FeCp_2BF_4$ and the isolated oil from the reaction of nickelacyclobutane (**1**) and Brookhart's acid with paramagnetic settings.

3. Spectra of new compounds

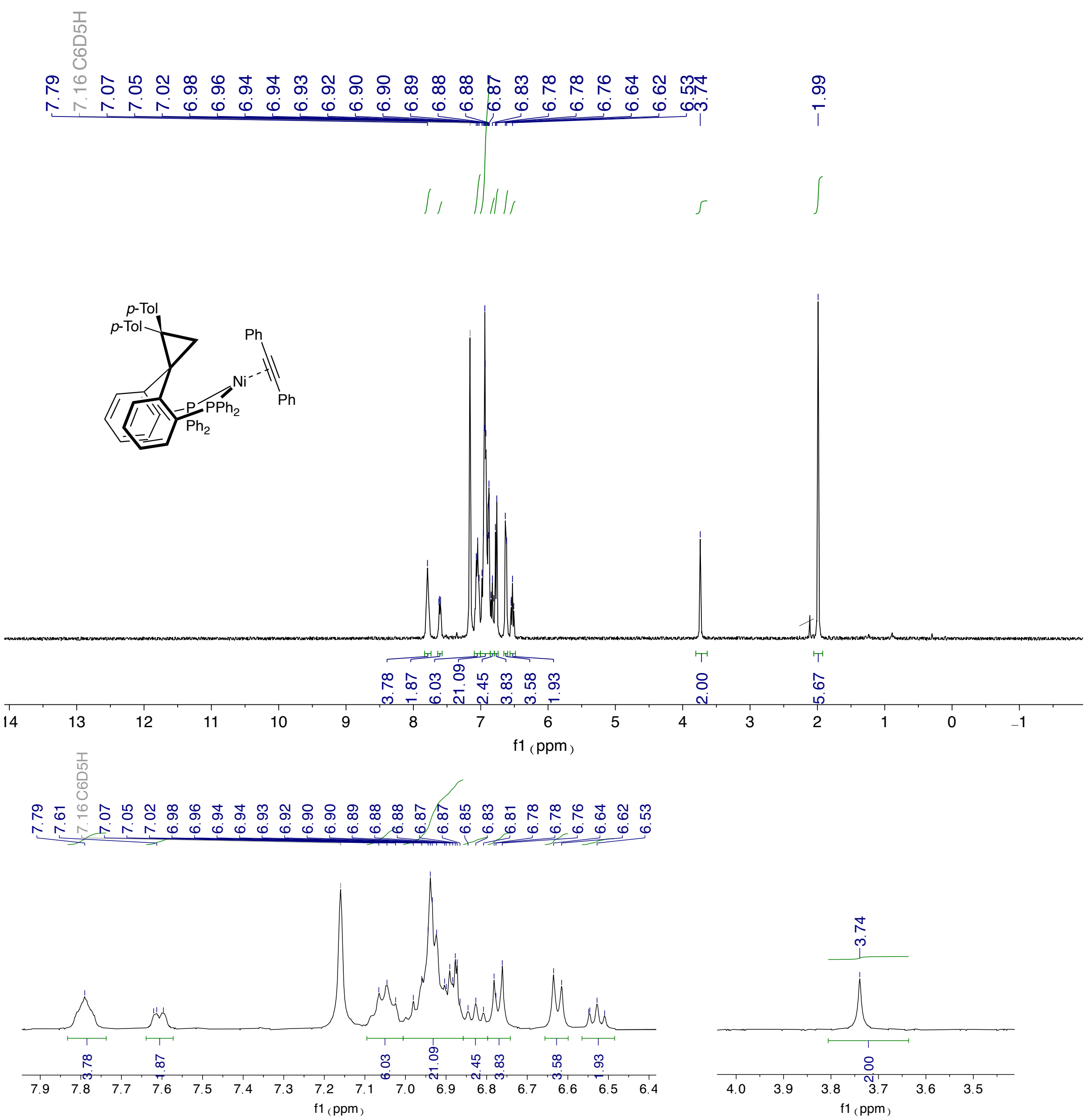

Figure S15. ^{1}H NMR of cyclopropane **5** in C_6D_6 at 25 ºC.

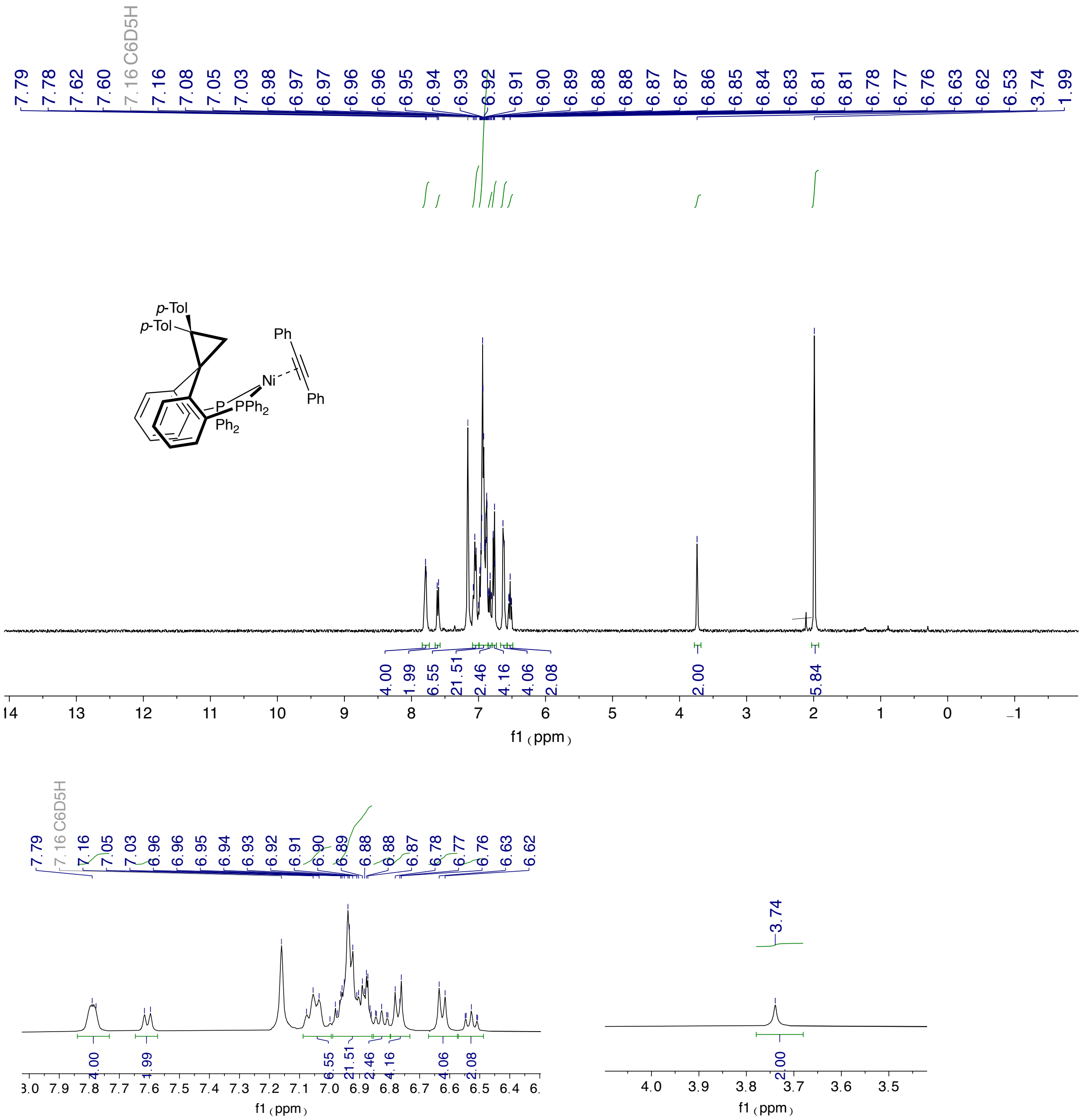

Figure S16. $^{1}H\{^{31}P\}$ NMR of cyclopropane **5** in C_6D_6 at 25 ºC.

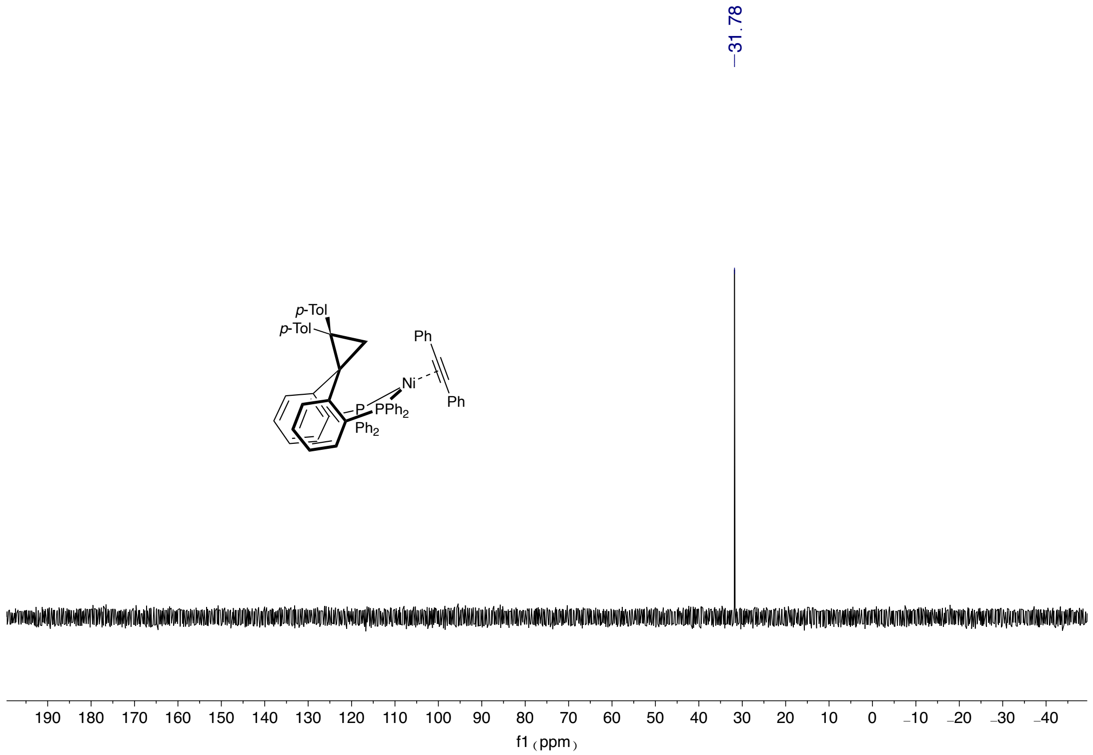

Figure S17. $^{31}P\{^{1}H\}$ NMR of cyclopropane **5** in C_6D_6 at 25 ºC.

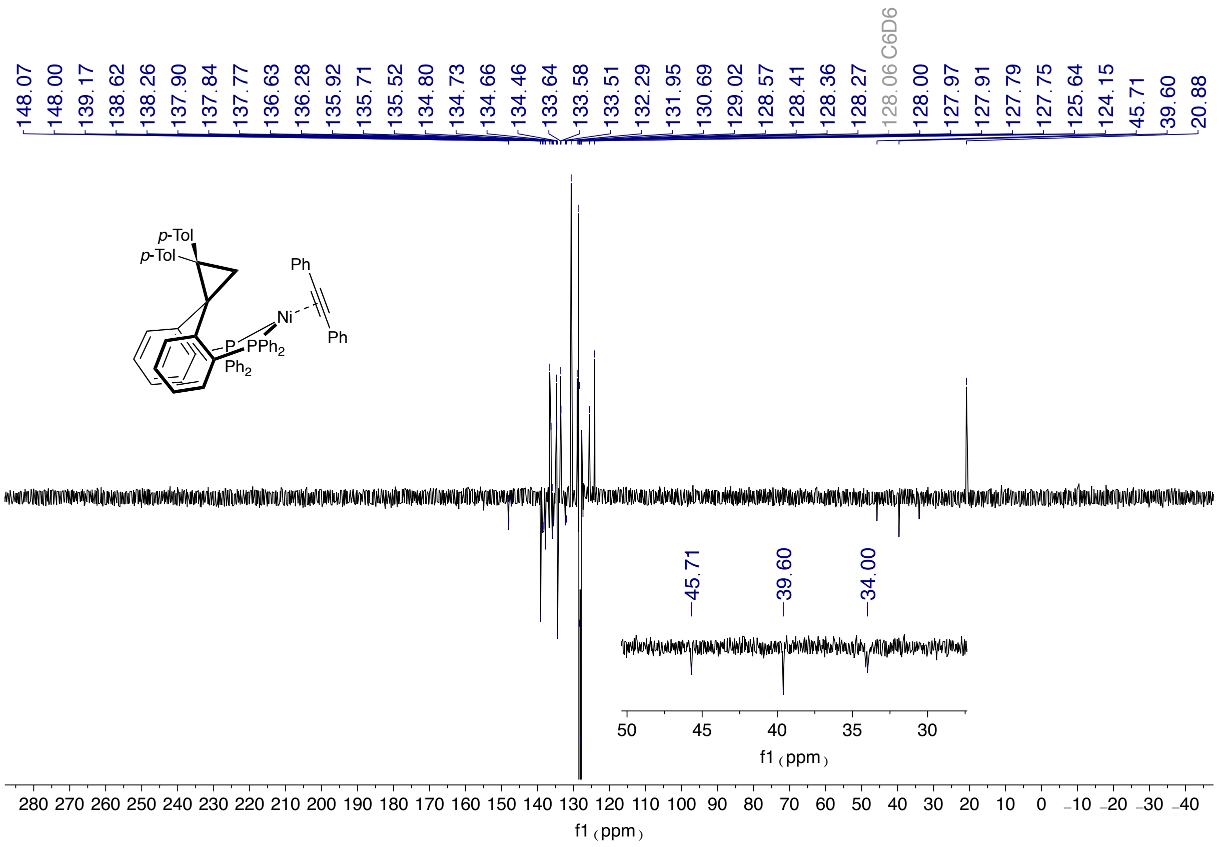

Figure S18. APT ^{13}C NMR of cyclopropane **5** in C_6D_6 at 25 ºC.

Spectrum

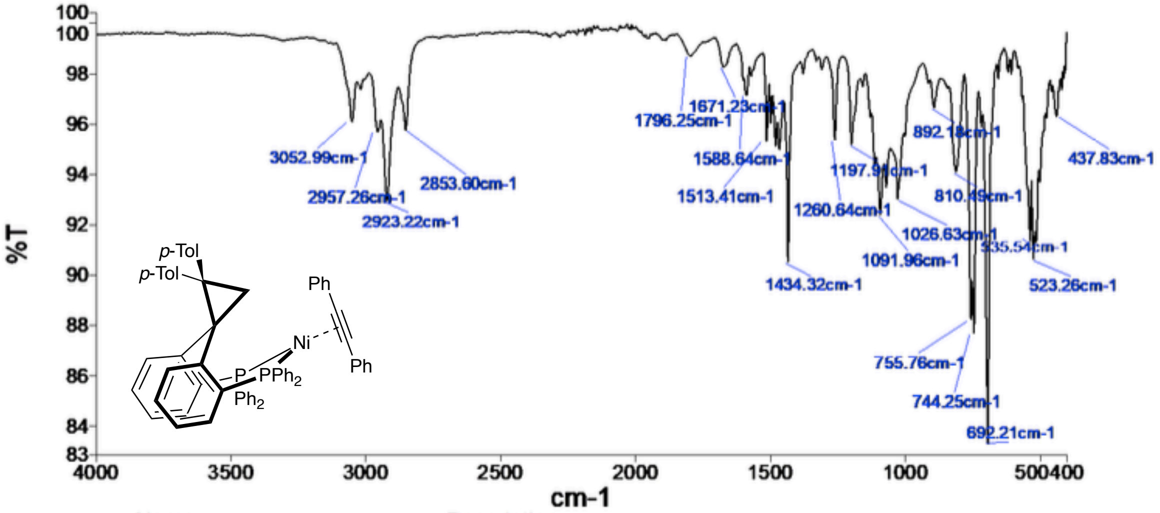

Figure S19. ATR-IR of cyclopropane **5** at 25 ºC.

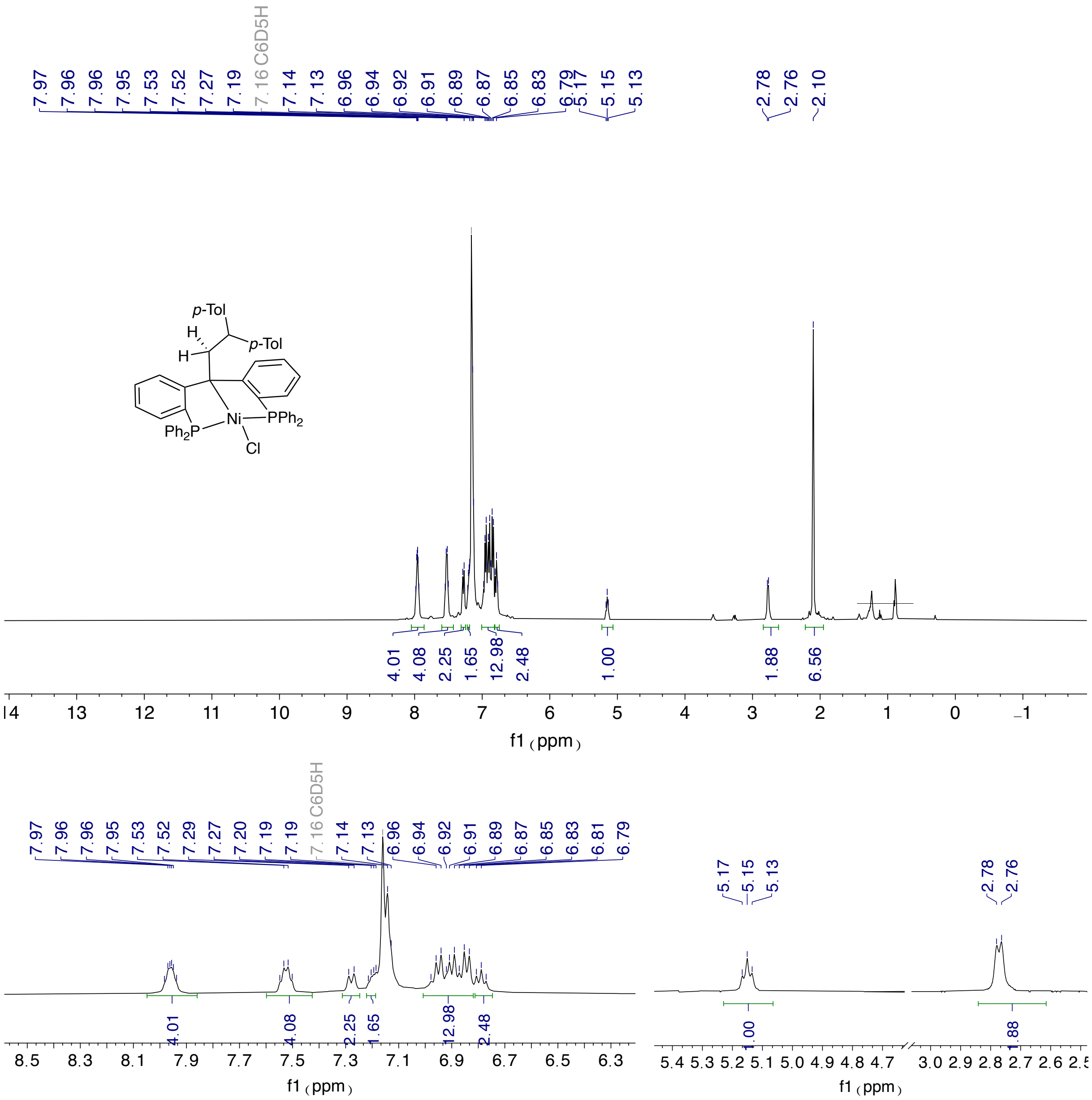

Figure S20. ^{1}H NMR of complex **7** in C_6D_6 at 25 ºC. Crossed peaks correspond to residual hexane.

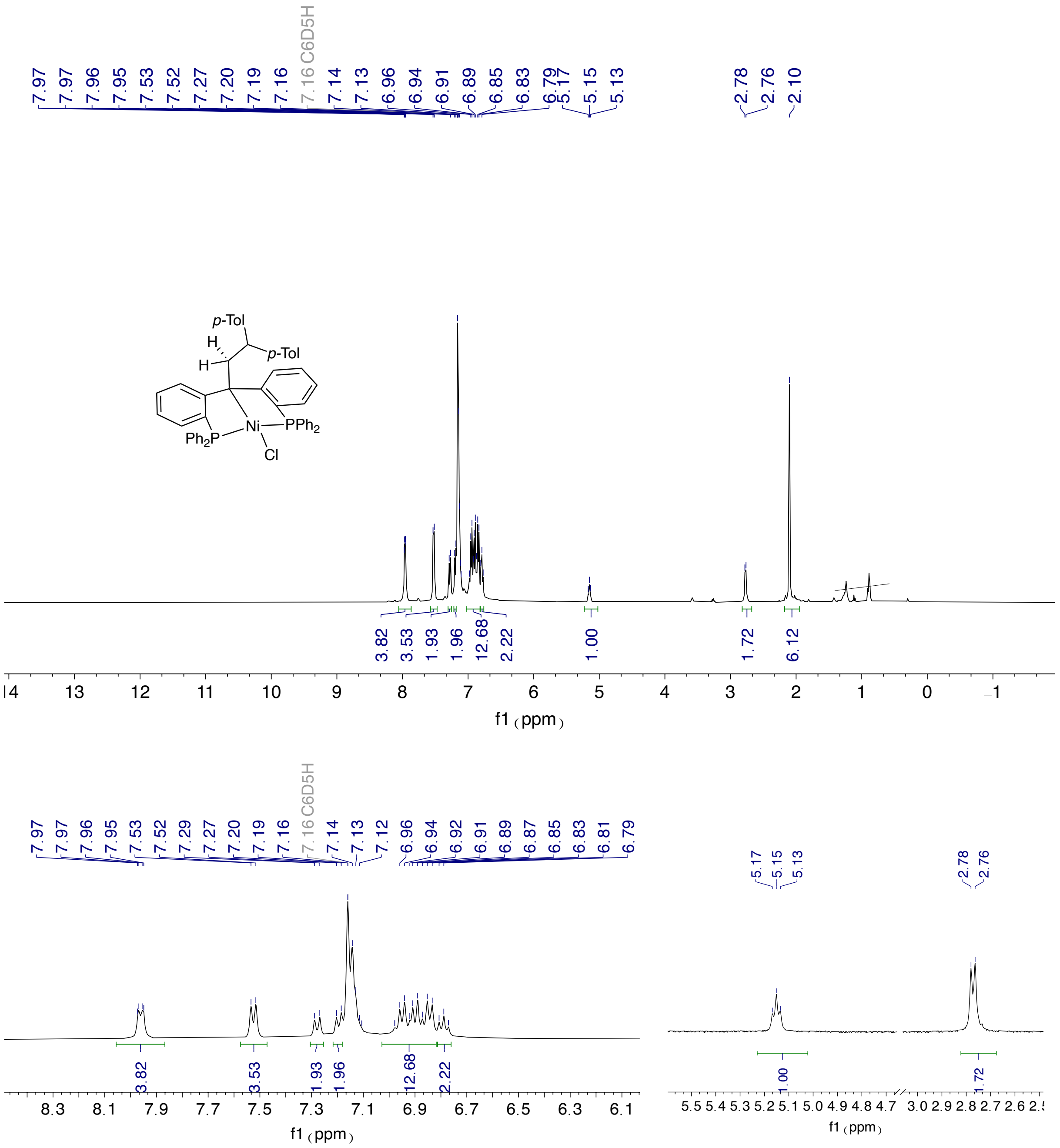

Figure S21. $^{1}H\{^{31}P\}$ NMR of complex **7** in C_6D_6 at 25 ºC. Crossed peaks correspond to residual hexane.

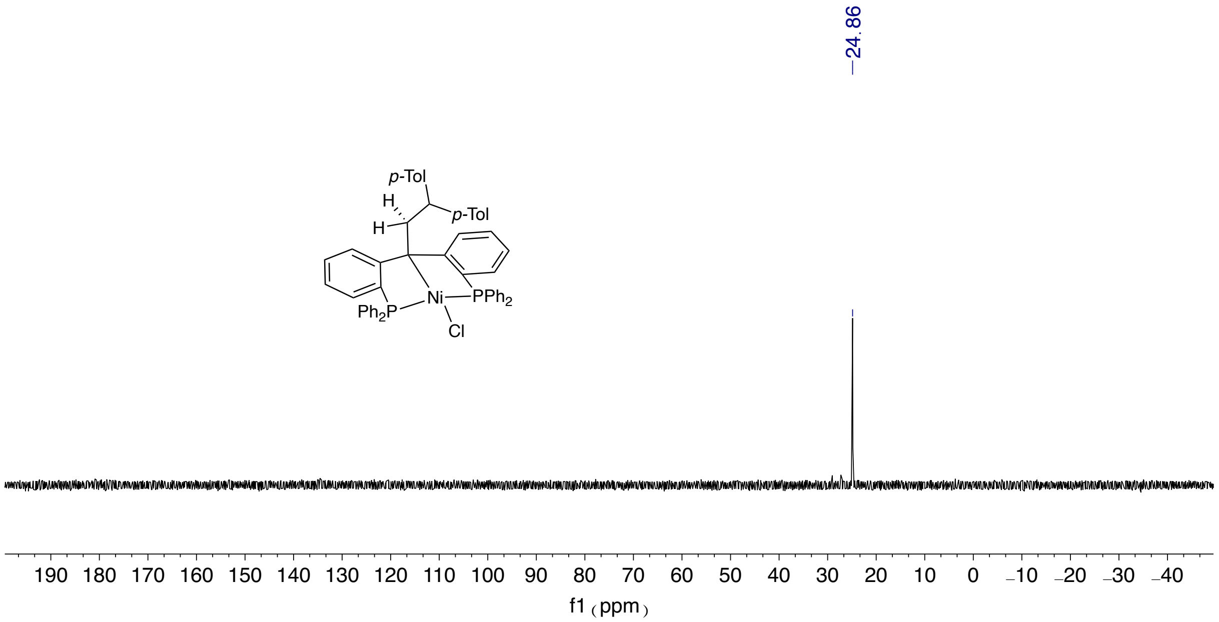

Figure S22. $^{31}P\{^{1}H\}$ NMR of complex **7** in C_6D_6 at 25 ºC.

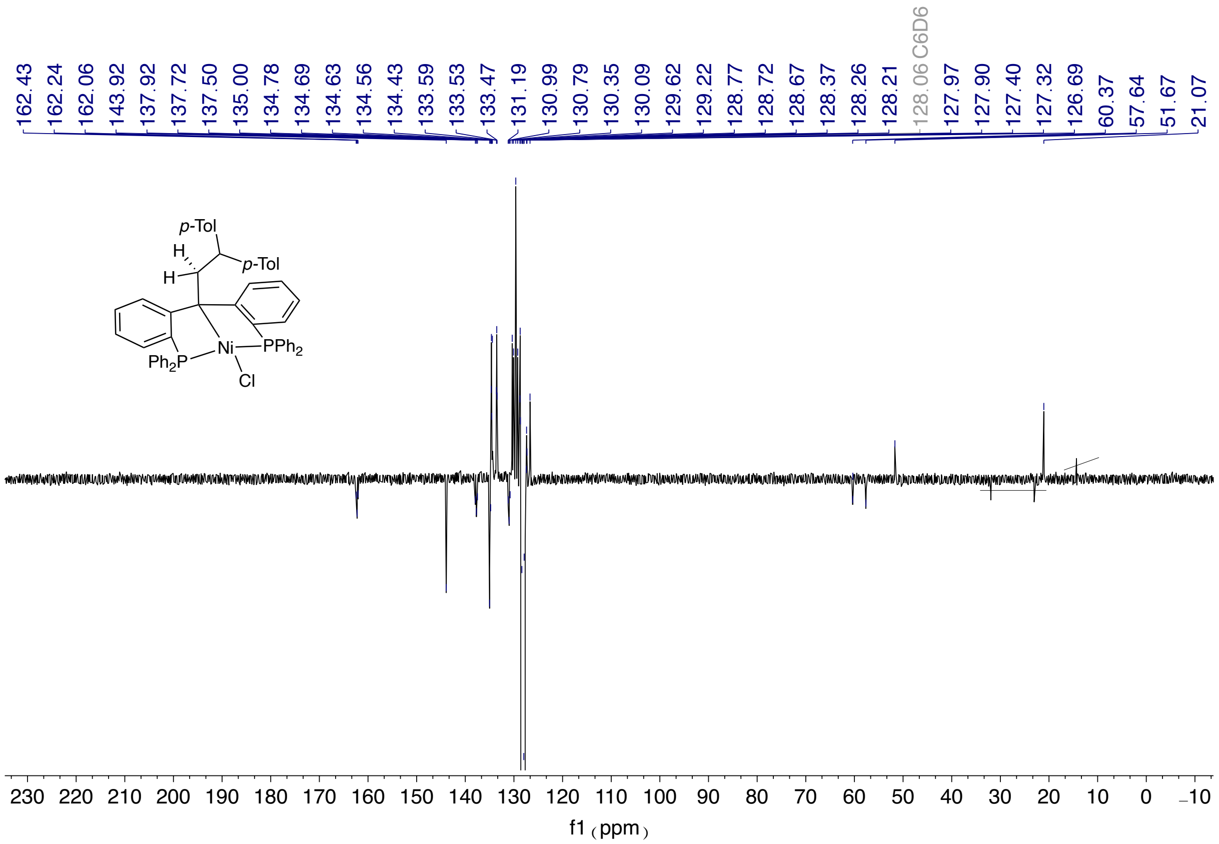

Figure S23. ^{13}C APT of complex **7** in C_6D_6 at 25 ºC. Crossed peaks correspond to residual hexane.

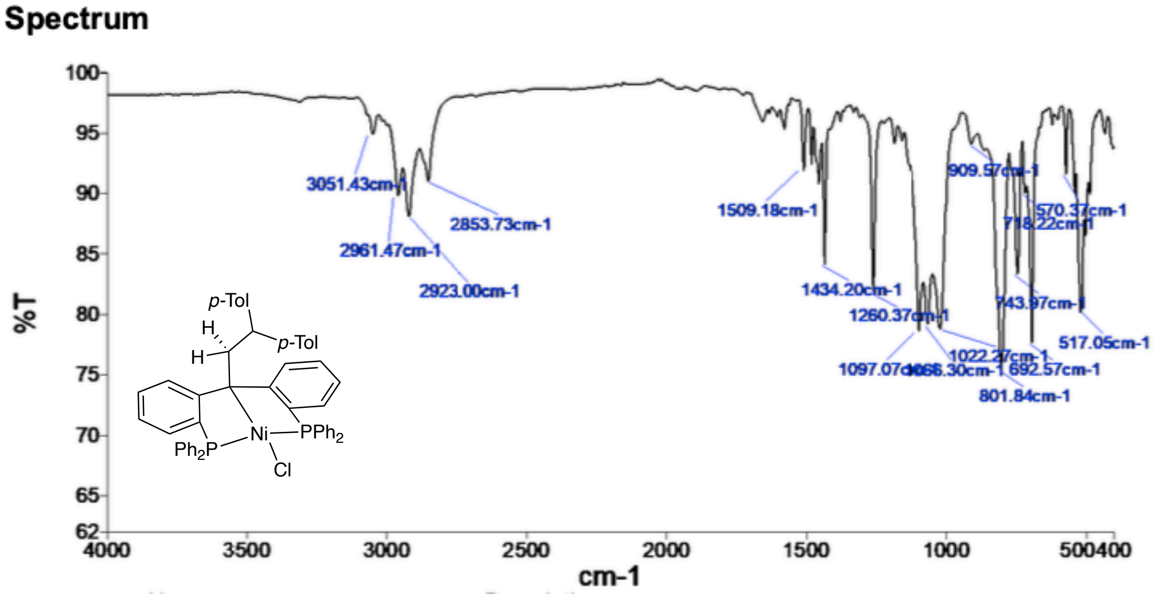

Figure S24. ATR-IR of complex **7** in C_6D_6 at 25 ºC.

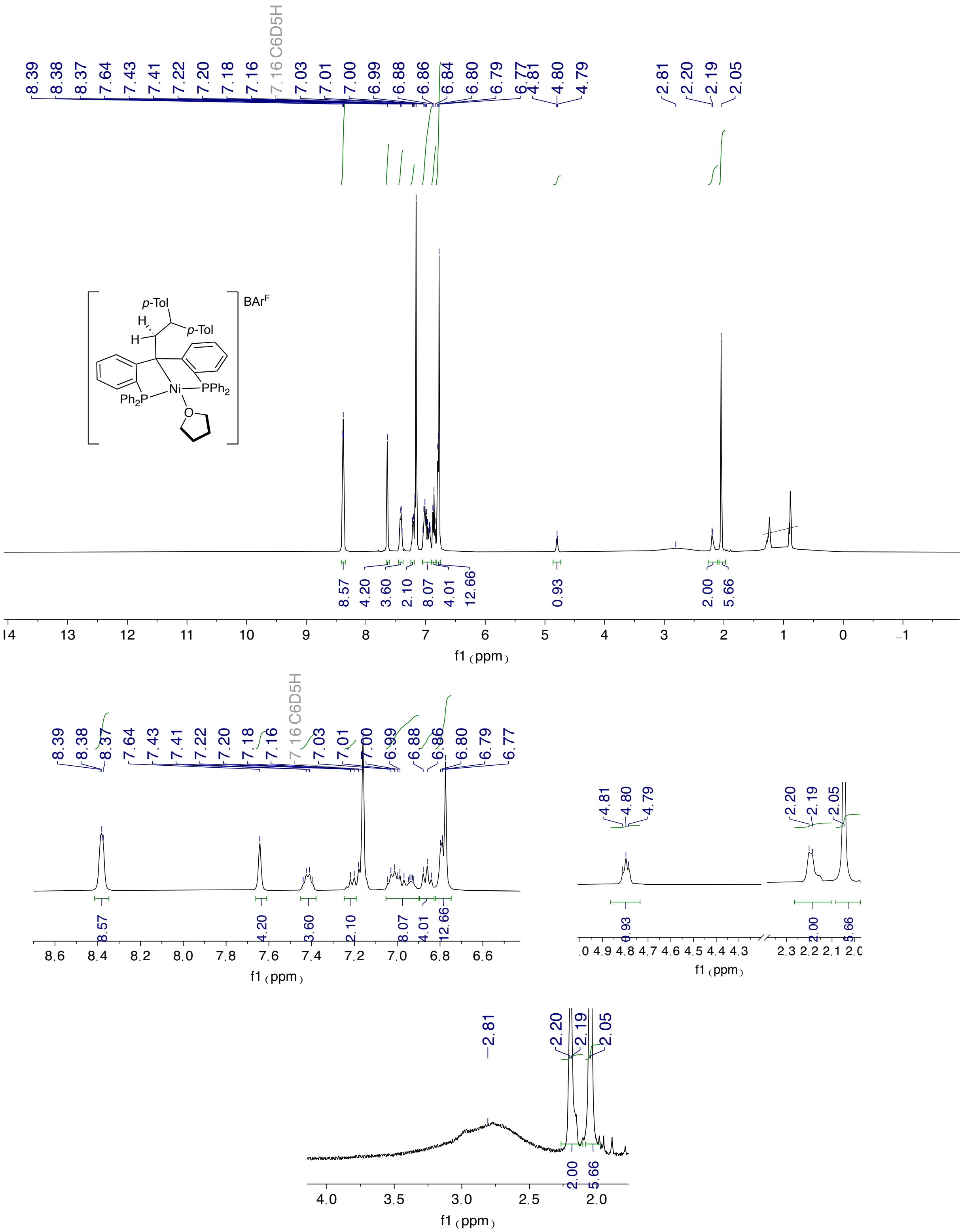

Figure S25. ^{1}H NMR of complex **8** in C_6D_6 at 25 ºC. Crossed peaks correspond to residual hexane.

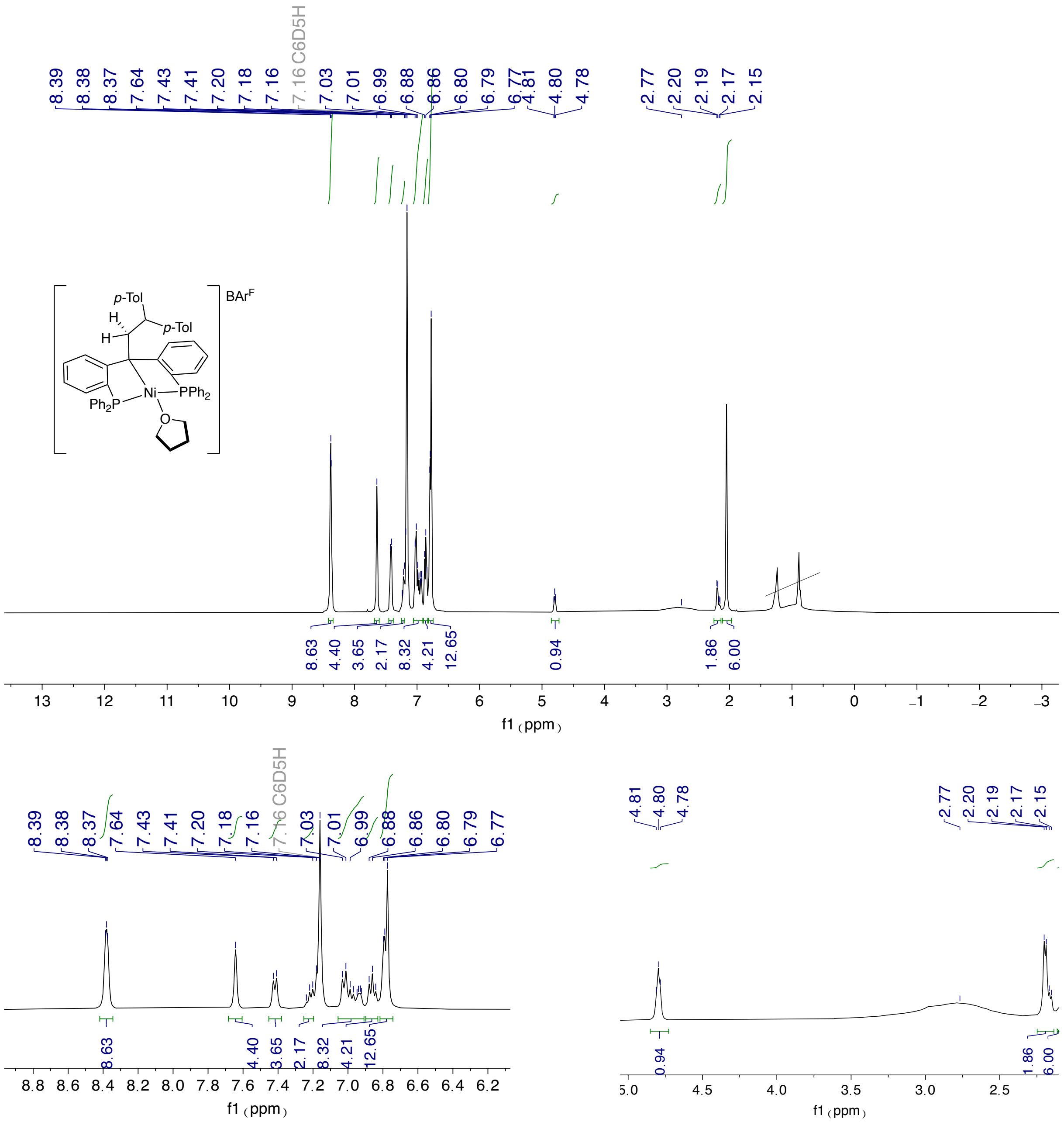

Figure S26. $^1H\{^{31}P\}$ NMR of complex **8** in C_6D_6 at 25 ºC. Crossed peaks correspond to residual hexane.

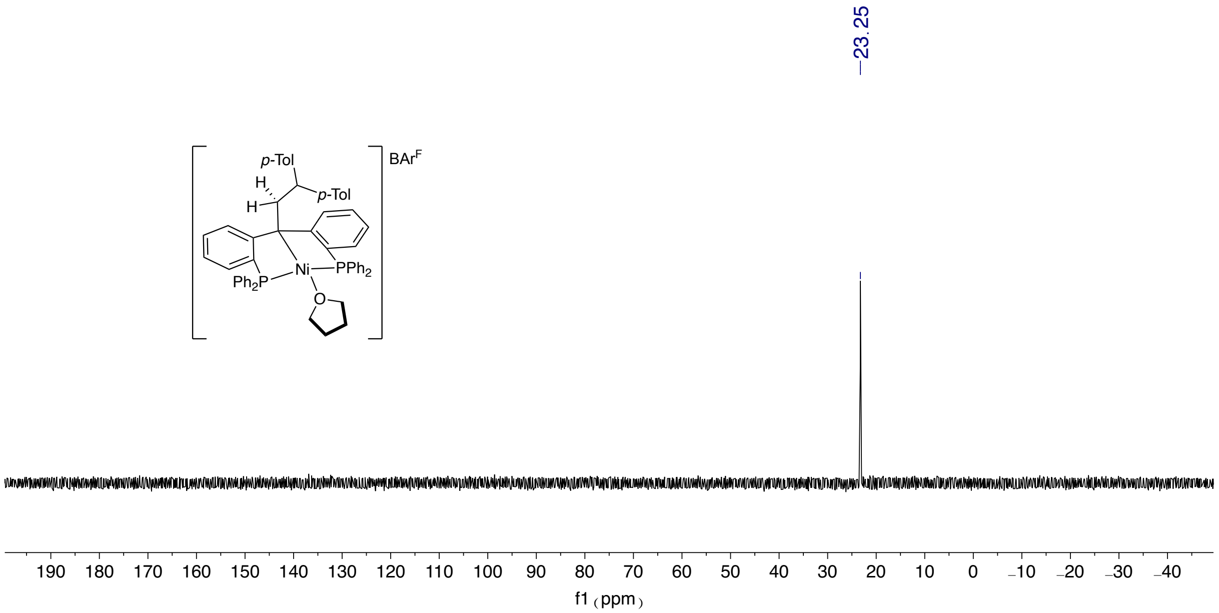

Figure S27. $^{31}P\{^{1}H\}$ NMR complex **8** in C_6D_6 at 25 ºC.

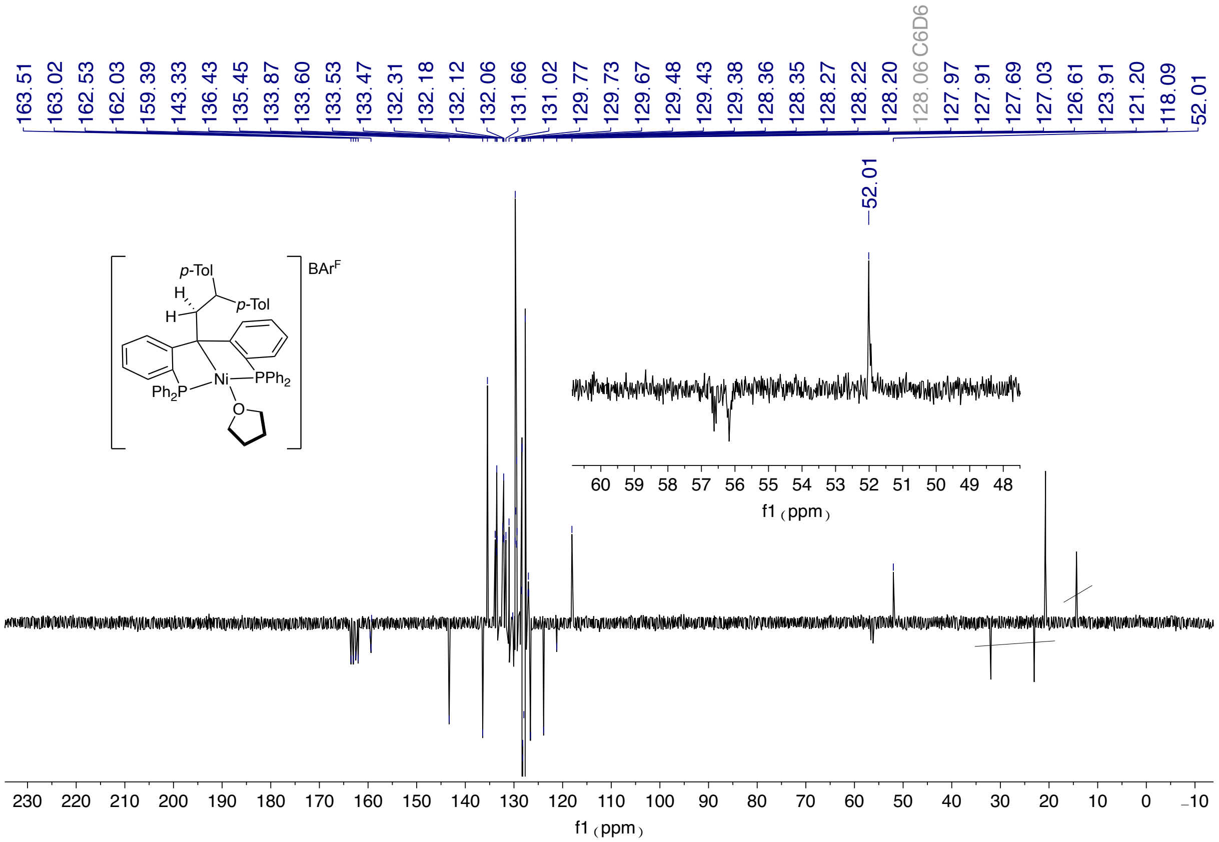

Figure S28. ^{13}C APT of complex **8** in C_6D_6 at 25 ºC. Crossed peaks correspond to residual hexane.

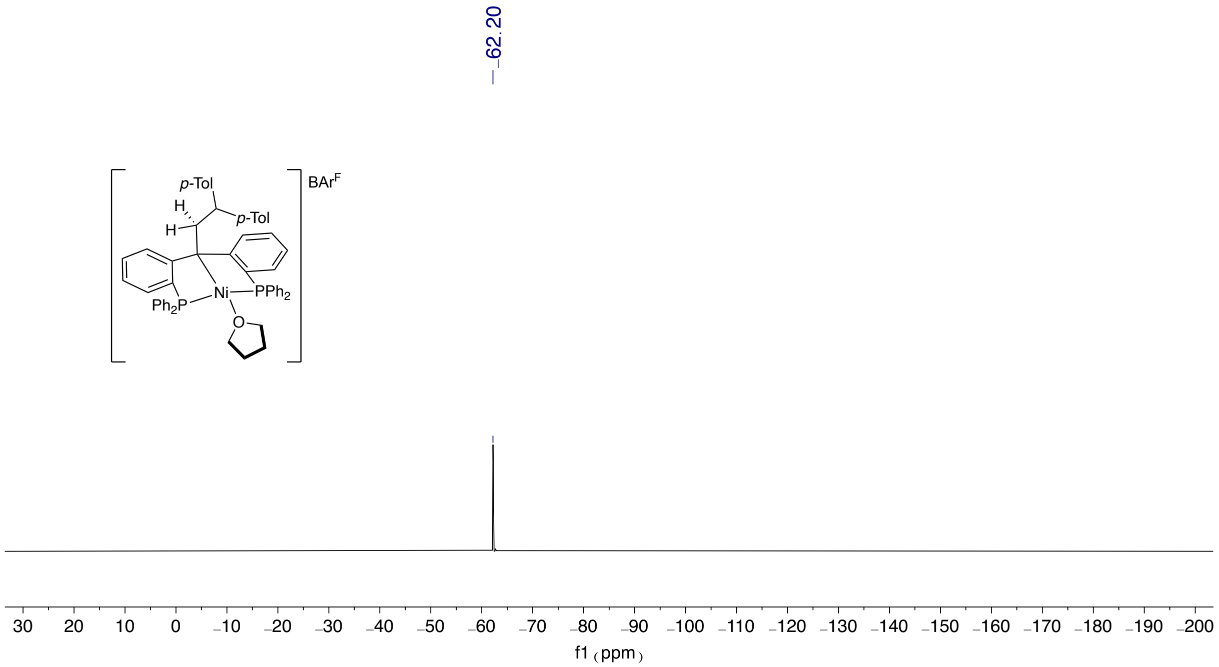

Figure S29. ^{19}F NMR of complex **8** in C_6D_6 at 25 ºC.

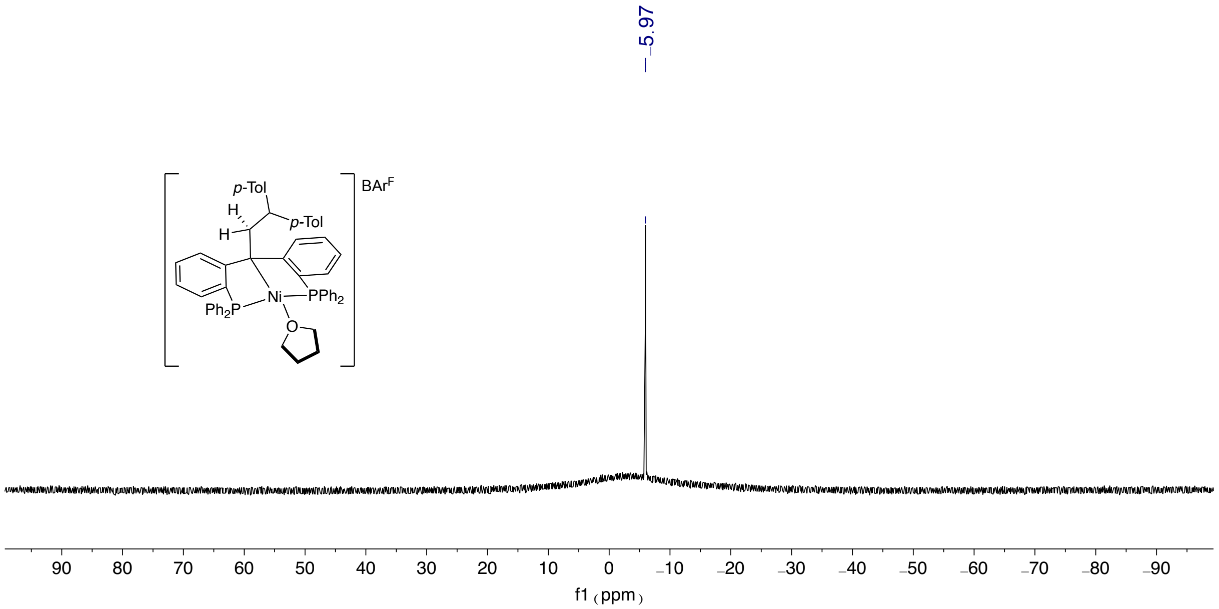

Figure S30. ^{11}B NMR of complex **8** in C_6D_6 at 25 ºC.

Spectrum

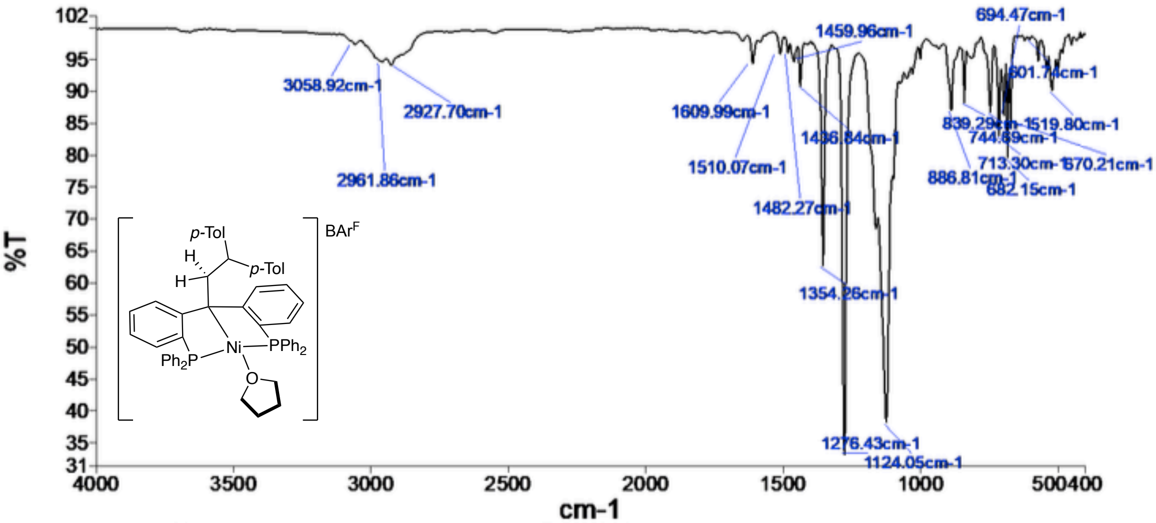

Figure S31. ATR-IR of complex **8** in C_6D_6 at 25 ºC.

4. X-ray crystal structure determinations

[P(cyclopropane)P]Ni(DPA) (5): $C_{67}H_{54}NiP_2 \cdot 0.5(C_7H_8)$, Fw = 1025.82, yellow needle, 0.41 × 0.07 × 0.04 mm^3, monoclinic, $P2_1/c$ (no. 14), a = 28.5835(16), b = 8.8001(4), c = 22.6627(12) Å, β = 111.347(3) °, V = 5309.5(5) $Å^3$, Z = 4, Dx = 1.283 g/cm^3, μ = 0.47 mm^{-1}. The diffraction experiment was performed on a Bruker Kappa ApexII diffractometer with sealed tube and Triumph monochromator (λ = 0.71073 Å) at a temperature of 150(2) K up to a resolution of $(\sin \theta/\lambda)_{max}$ = 0.65 $Å^{-1}$. The Eval15 software[8] was used for intensity integration. A multi-scan absorption correction and scaling was performed with SADABS[9] (correction range 0.65-0.75). A total of 91166 reflections was measured, 12211 reflections were unique (Rint = 0.106), 7902 reflections were observed [I>2σ(I)]. The structure was solved with Patterson superposition methods using SHELXT.[10] Structure refinement was performed with SHELXL-2018[11] on F^2 of all reflections. Non-hydrogen atoms were refined freely with anisotropic displacement parameters. The toluene solvent molecule was disordered on an inversion center (occupancy ½). Hydrogen atoms of the metal complex were located in difference Fourier maps. Hydrogen atoms of the toluene molecule were introduced in calculated positions. The hydrogen atoms at C38 were refined freely with isotropic displacement parameters. All other hydrogen atoms were refined with a riding model. 704 Parameters were refined with 123 restraints (geometry and flatness in the disordered toluene). R1/wR2 [I > 2σ(I)]: 0.0517 / 0.1008. R1/wR2 [all refl.]: 0.0961 / 0.1160. S = 1.024. Residual electron density between -0.34 and 0.39 e/$Å^3$. Geometry calculations and checking for higher symmetry were performed with the PLATON program.[12]

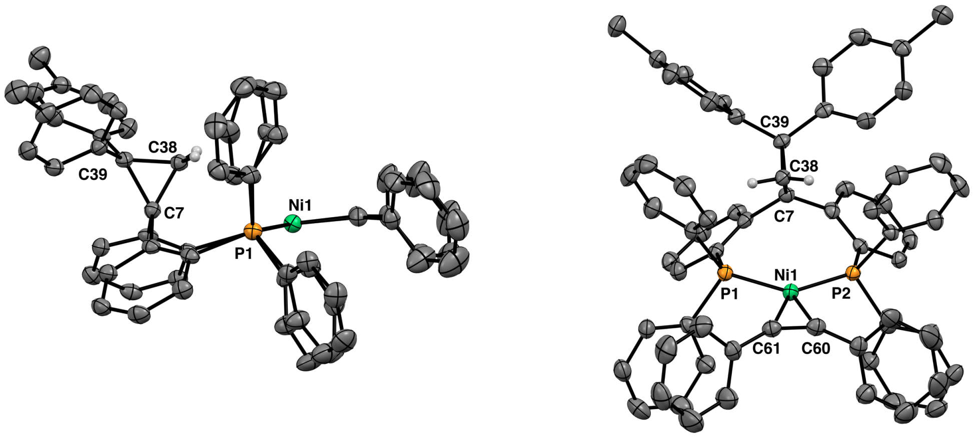

Figure S32. Two views of the molecular structure of **5** in the crystal. Displacement ellipsoids are drawn at the 50% probability level. Solvent molecules and most H atoms are omitted for clarity. Selected bond lengths (Å): Ni1–P1 2.1494(7), Ni1–P2 2.1601(7), Ni1–C60 1.898(3), Ni1–C61 1.911(3), C60–C61 1.279(4), C7–C38 1.503(3), C7–C39 1.590(3), C38–C39 1.506(4).

[PC(CH$_2$CH(*p*-Tol)$_2$)P]NiCl (7): $C_{53}H_{45}ClNiP_2$, Fw = 837.99, red plate, 0.34 × 0.23 × 0.06 mm^3, triclinic, P$\overline{1}$ (no. 2), a = 11.6704(5), b = 15.0005(9), c = 23.5964(12) Å, α = 89.247(3), β = 89.659(2), γ = 88.484(2) °, V = 4129.0(4) Å^3, Z = 4, D_x = 1.348 g/cm^3, μ = 0.65 mm^{-1}. The diffraction experiment was performed on a Bruker Kappa ApexII diffractometer with sealed tube and Triumph monochromator (λ = 0.71073 Å) at a temperature of 150(2) K up to a resolution of $(\sin \theta/\lambda)_{max}$ = 0.61 Å^{-1}. The crystal appeared to be twinned with a twofold rotation about *uvw* = [1,0,0] as twin operation. Consequently, two orientation matrices were used for the intensity integration with the Eval15 software.[8] resulting in a HKLF5-file.[13] A multi-scan absorption correction and scaling was performed with TWINABS[14] (correction range 0.52-0.75). A total of 88276 reflections was measured, 15370 reflections were unique (R_{int} = 0.110), 9163 reflections were observed [$I>2\sigma(I)$]. The structure was solved with Patterson superposition methods using SHELXT.[10] Structure refinement was performed with SHELXL-2018[11] on F^2 of all reflections. Non-hydrogen atoms were refined freely with anisotropic displacement parameters. Hydrogen atoms were introduced in calculated positions and refined with a riding model. 1032 Parameters were refined with no restraints. R1/wR2 [$I > 2\sigma(I)$]: 0.0668 / 0.1475. R1/wR2 [all refl.]: 0.1286 / 0.1756. S = 1.030. Residual electron density between -0.69 and 1.10 e/Å^3. Twin fraction BASF = 0.5187(16). Geometry calculations and checking for higher symmetry was performed with the PLATON program.[12]

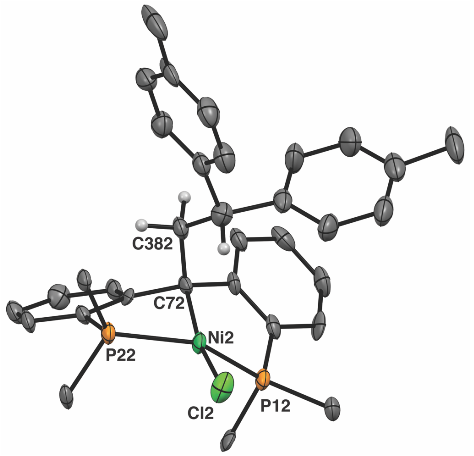

Figure S33. Molecular structure of **7**. Displacement ellipsoids are drawn at the 50% probability level. Only one of the two independent molecules is shown. Solvent molecules, most H atoms and phenyl rings from the phosphines are omitted for clarity. Selected bond lengths (Å) and angles (°): Ni2–P22 2.1853(16), Ni2–P12 2.1562(15), Ni2–Cl2 2.2245(18), Ni2–C72 2.031(6), C72–C382 1.539(7), P12–Ni2–P22 145.87(7), C72–Ni2–Cl2 165.75(16), C72–Ni2–P12 86.35(16), P12–Ni2–Cl2 98.97(7), P22–Ni2–Cl2 97.72(7), P22–Ni2–C72 84.67(16).

{[PC(CH$_2$CH(*p*-Tol)$_2$)P]Ni(THF)} BAr^F (9): [$C_{57}H_{53}NiOP_2$]($C_{32}H_{12}BF_{24}$) · C_6H_{14}, Fw = 1824.04, red needle, 0.55 × 0.14 × 0.08 mm^3, triclinic, $P\overline{1}$ (no. 2), a = 12.8777(7), b = 17.1363(6), c = 20.3499(10) Å, α = 77.764(2), β = 76.568(3), γ = 86.985(3) °, V = 4268.6(4) Å^3, Z = 2, D_x = 1.419 g/cm^3, μ = 0.37 mm^{-1}. The diffraction experiment was performed on a Bruker Kappa ApexII diffractometer with sealed tube and Triumph monochromator (λ = 0.71073 Å) at a temperature of 150(2) K up to a resolution of $(\sin \theta/\lambda)_{max}$ = 0.65 Å^{-1}. The Eval15 software[8] was used for intensity integration. A numerical absorption correction and scaling was performed with SADABS[14] (correction range 0.83-1.00). A total of 63405 reflections was measured, 19594 reflections were unique (R_{int} = 0.053), 12556 reflections were observed [$I>2\sigma(I)$]. The structure was solved with Patterson superposition methods using SHELXT.[10] Structure refinement was performed with SHELXL-2018[11] on F^2 of all reflections. Non-hydrogen atoms were refined freely with anisotropic displacement parameters. Five of the CF_3 groups were refined with a disorder model. The hydrogen atoms of the metal complex were located in difference Fourier maps. All other hydrogen atoms were introduced in calculated positions. All hydrogen atoms were refined with a riding model. 1256 Parameters were refined with 3078 restraints (distances, angles and displacement parameters in the disordered CF_3 moieties). R1/wR2 [$I > 2\sigma(I)$]: 0.0511 / 0.1201. R1/wR2 [all refl.]: 0.0918 / 0.1351. S = 1.054. Residual electron density between -0.39 and 0.54 e/Å^3. Geometry calculations and checking for higher symmetry was performed with the PLATON program.[12]

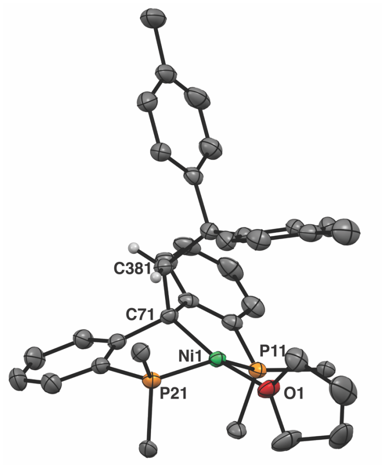

Figure S34. Molecular structure of **9**. Displacement ellipsoids are drawn at the 50% probability level. BAr^F anion, solvent molecules, most H atoms, and phenyl rings from the phosphines are omitted for clarity. Selected bond lengths (Å) and angles (º): Ni1–P21 2.1948(6), Ni1–P11 2.1625(7), Ni–O1 1.9973(16), Ni1–C71 1.998(2), C71–C381 1.560(3), P11–Ni1–P12 146.43(3), O1–Ni1–C71 168.24(8), P21–Ni1–C71 86.32(7), C71–Ni1–P11 86.33(7), P11–Ni1–O1 98.41(5), O1–Ni1–P21 95.33(5).

5. DFT calculations

5.1 General information

DFT calculations were performed using the Gaussian 16 software package version C.01.[15] Geometry optimizations were carried out in vacuum at the B3LYP-GD3BJ/6-31g(d,p) level of theory on all atoms. Frequency analyses on all stationary points were used to ensure that they are minima (no imaginary frequency) or transition states (one imaginary frequency). Transition states were calculated using the QST3 (synchronous transit-guided quasi-Newton number 3) method or using the opt=TS (Berny algorithm) keyword. The guess structures used as starting point for TS calculations were based on the results of relaxed potential energy surface scans (PES). ΔG^{o} was calculated by single point calculation at the B3LYP-GD3BJ/def2TZVP/SMD level of theory using THF, toluene or benzene as solvent as indicated. Thermal correction was obtained at the B3LYP-GD3BJ/6-31g(d,p) level of theory with temperature 298.15 K and pressure 1 atmosphere. Broken symmetry calculation of **10-H-open** (no sym) as an open-shell singlet was performed by using guess=(mix,NoSymm) keywords in the optimization.

5.2 Reactivity with H_2

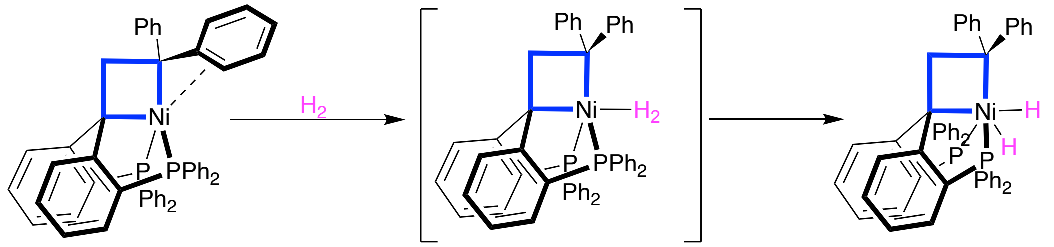

10
0 kcal/mol

10-H₂
8.6 kcal/mol

17
24.3 kcal/mol

Scheme S1. Gibbs free energy profiles oxidative addition of H_2. Calculations were computed at the B3LYP-GD3BJ/def2TZVP/SMD(toluene)//B3LYP-GD3BJ/6-31g(d,p) level of theory.

5.3 Reactivity with terminal alkynes

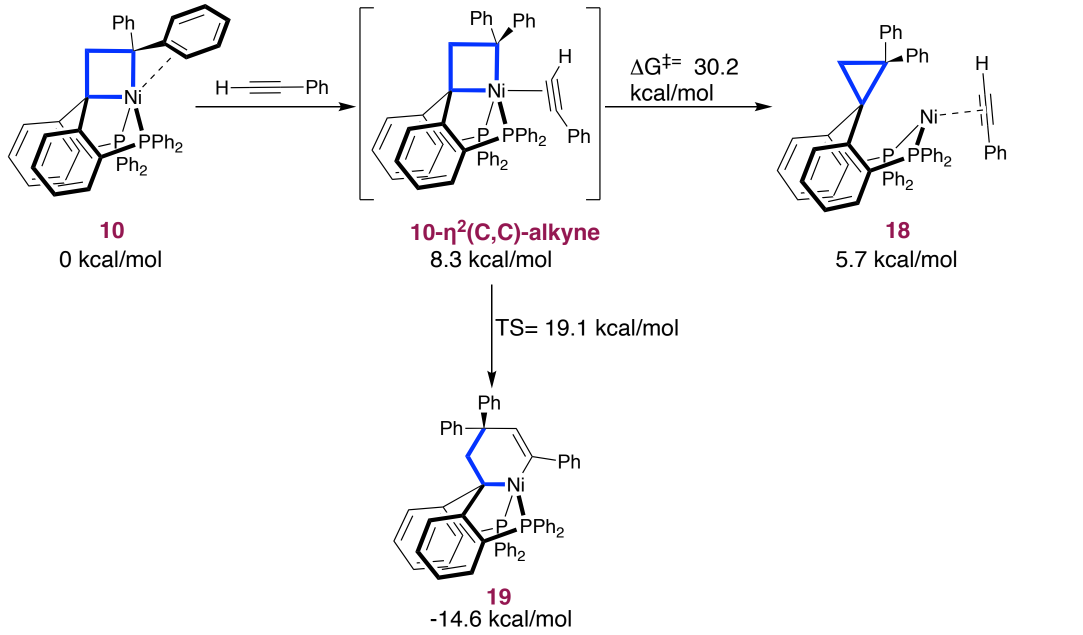

Scheme S2. Gibbs free energy profiles for different reactivity of **10** with phenylacetylene. Calculations were computed at the B3LYP-GD3BJ/def2TZVP/SMD(C_6D_6)//B3LYP-GD3BJ/6-31g(d,p) level of theory.

5.4 Methathesis after one electron oxidation of the nickelacyclobutane

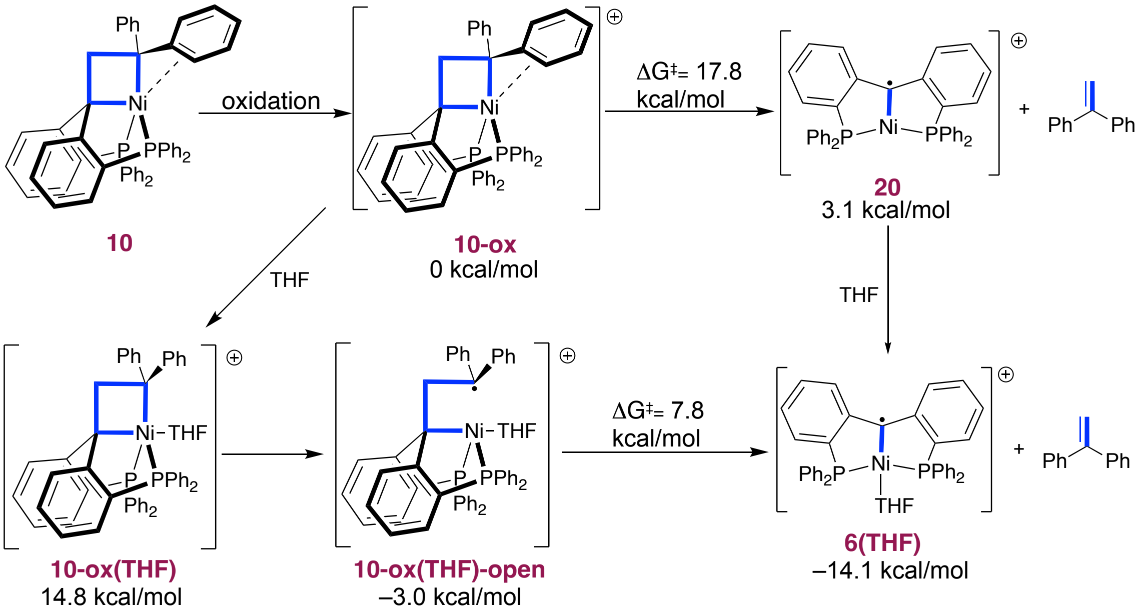

Scheme S3. Gibbs energy for the oxidation of the nickelacyclobutane calculated at the B3LYP-GD3BJ/def2TZVP/SMD(THF)//B3LYP-GD3BJ/6-31g(d,p) level of theory.

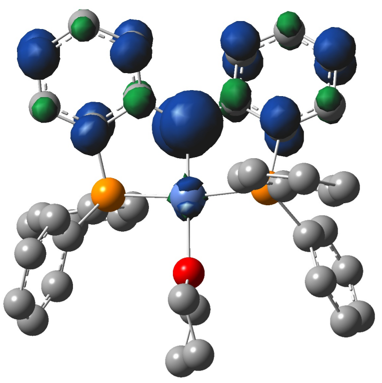

Figure S35. Spin density of complex 6(THF).

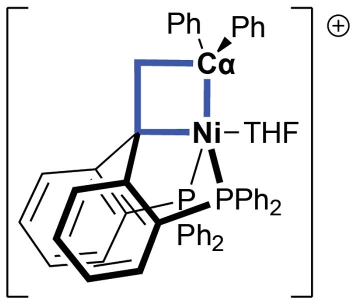

10-ox(THF)

Potential Energy Scan
Elongantion Ni-Cα 0.01 Å each step

B3LYP-GD3BJ/6-31g(d,p) level of theory

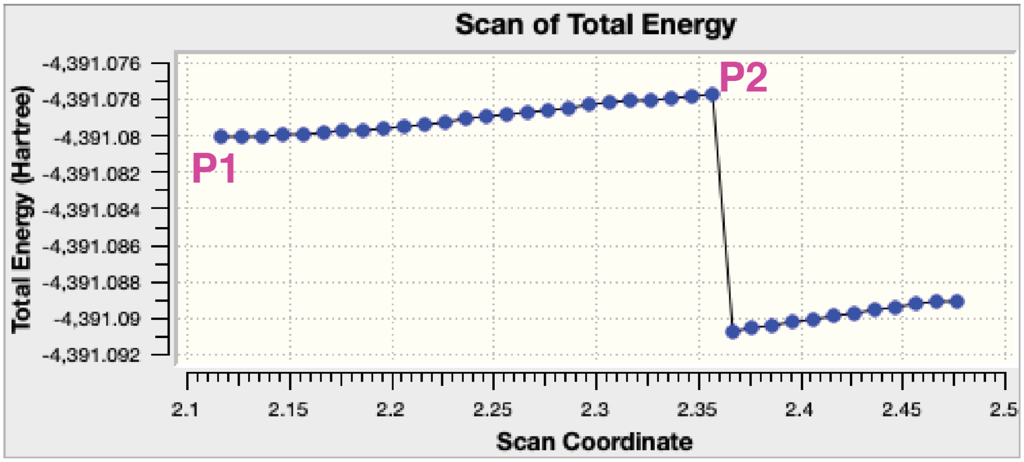

P1 [initial point, 10-ox(THF)]= -4391.080074 Hartree
P2 = -4391.077818 Hartree

P2 - P1= 0.002256 Hartree= 1.415 kcal/mol

Figure S36. Relaxed Potential Energy Surface scan of the elongation of bond Ni-Cα of 10-ox(THF).

5.5 Reactivity with Bronsted acids

5.4.1 HCl

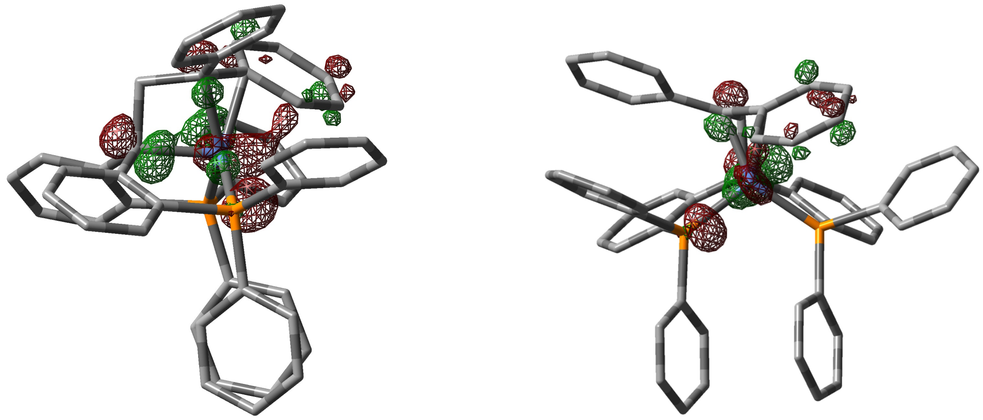

HOMO

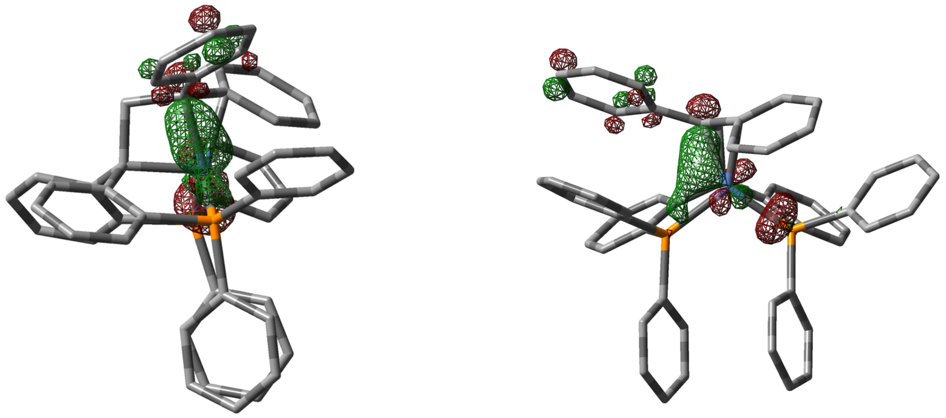

HOMO-1

Figure S37. Two views of the HOMO and HOMO-1 orbitals of **10** extracted from DFT calculations at the B3LYP-GD3BJ/def2TZVP//B3LYP-GD3BJ/6-31g(d,p) level of theory.

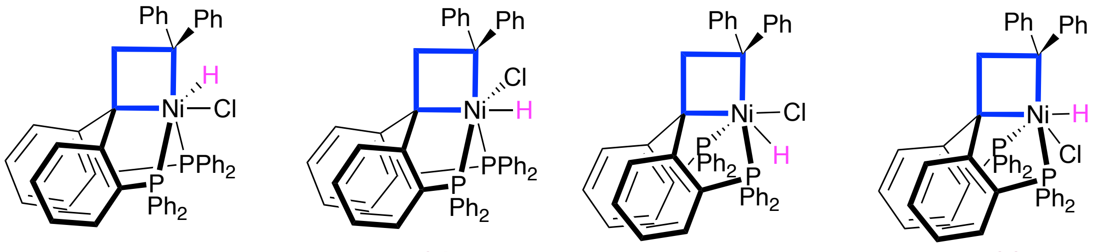

**13**	**21**	**22**	**23**
0.3 kcal/mol	13.2 kcal/mol	4.7 kcal/mol	18.5 kcal/mol

Figure S38. Gibbs free energies of the oxidative addition products from reactivity of **10** with HCl computed at the B3LYP-GD3BJ/def2TZVP/SMD(THF)//B3LYP-GD3BJ/6-31g(d,p) level of theory.

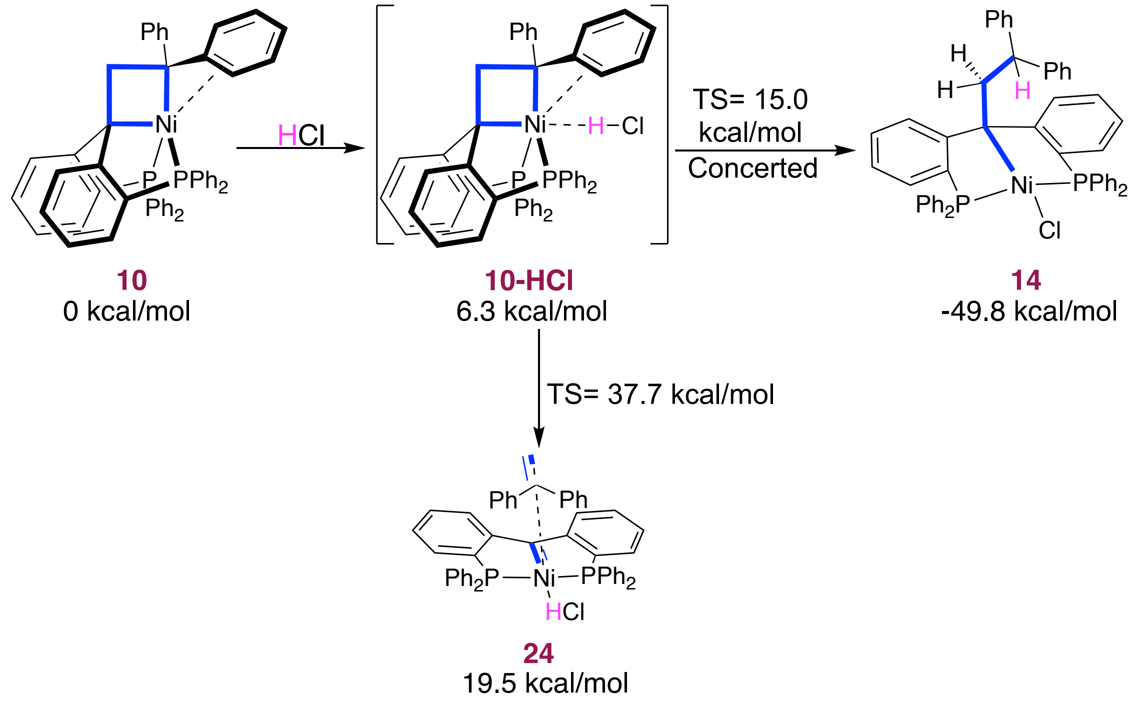

Scheme S4. Gibbs free energy profiles for different reactivity of **10-HCl**. Calculations were computed at the B3LYP-GD3BJ/def2TZVP/SMD(THF)//B3LYP-GD3BJ/6-31g(d,p) level of theory.

5.4.2 Brookhart's acid

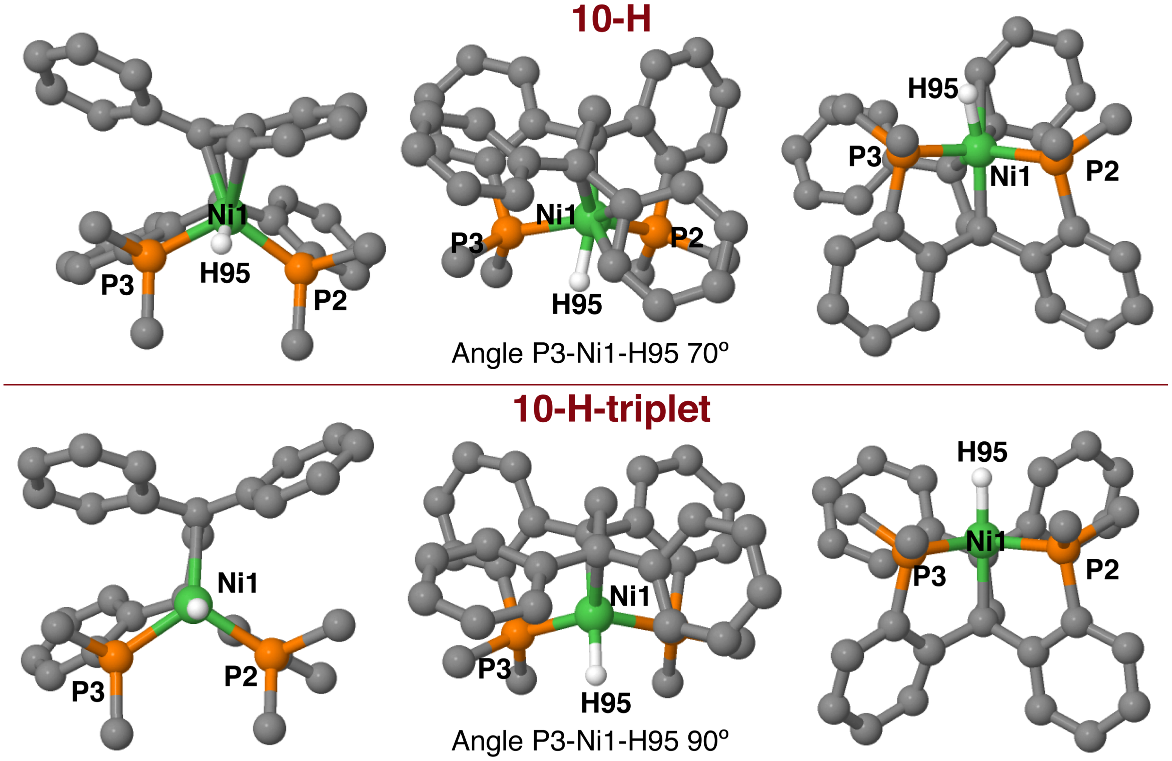

Figure S39. Optimized structures of **10-H** and **10-H-triplet** from different perspectives.

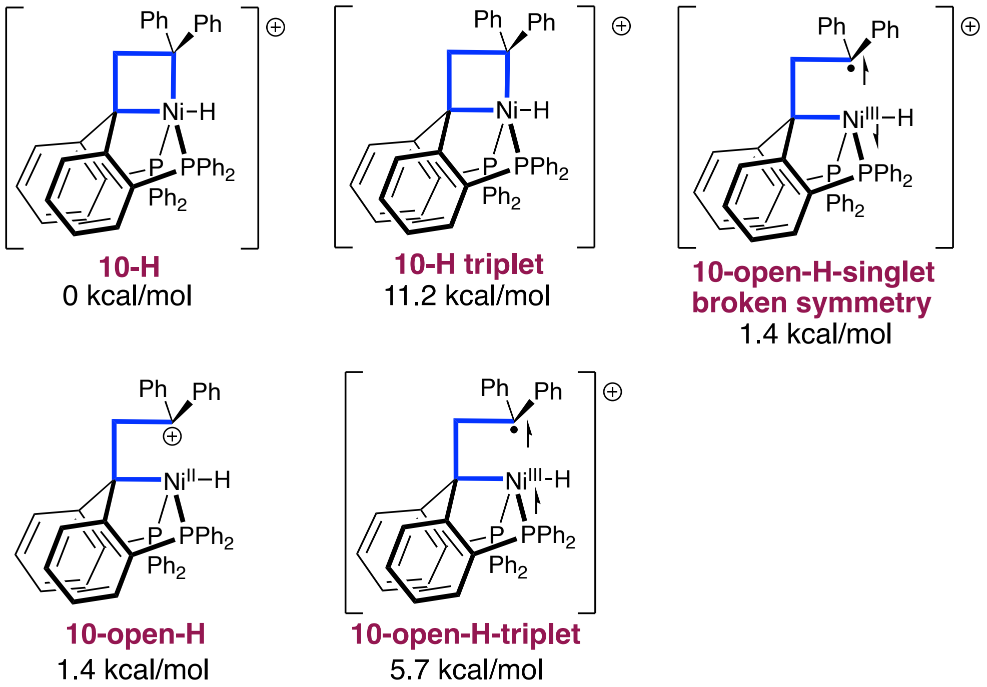

Figure S40. Gibbs free energies of **10-H** and other related structures computed at the B3LYP-GD3BJ/def2TZVP/SMD(THF)//B3LYP-GD3BJ/6-31g(d,p) level of theory.

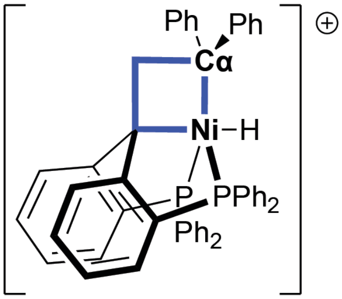

10-H

Potential Energy Scan
Elongantion Ni-Cα 0.01 Å each step

B3LYP-GD3BJ/6-31g(d,p) level of theory

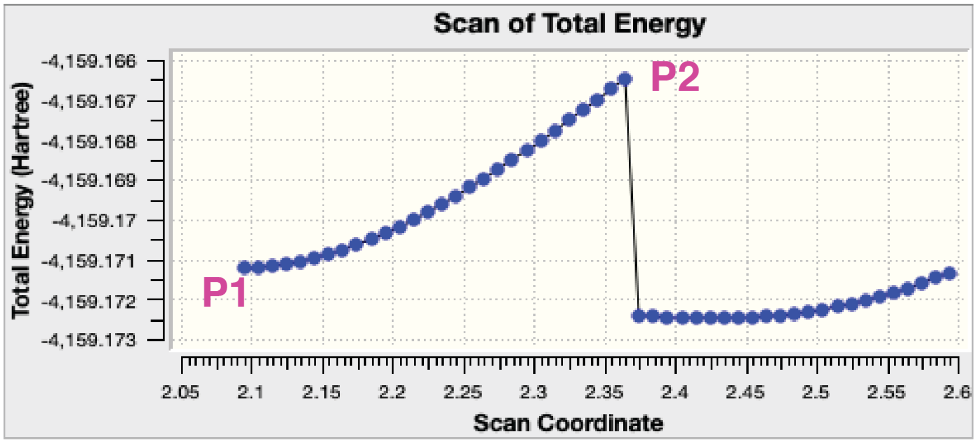

P1 (initial point, 10-H)= -4159.171228 Hartree
P2 = -4159.166465 Hartree

P2 - P1= 0.004763 Hartree= 2.988 kcal/mol

Figure S41. Relaxed Potential Energy Surface Scan of the elongation of bond Ni-Cα of 10-H.

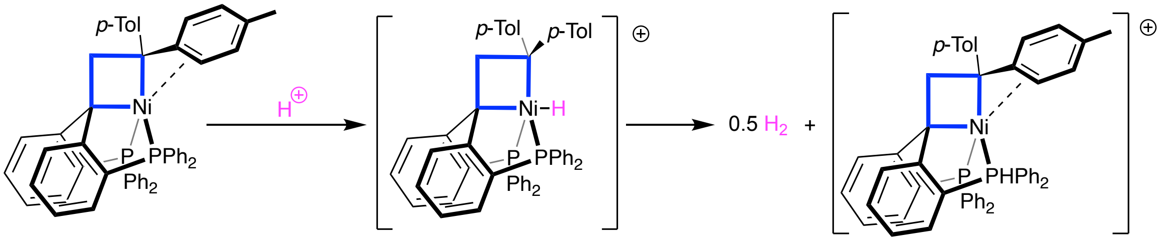

1 **1-H** **1-ox**

Scheme S5. Proposed reactivity of protonation of nickelacyclobutane **1** involving a Ni(III) intermediate.

5.6 Table of energies

Table S1. Single point energies obtained at the B3LYP-GD3BJ/DEF2TZVP/SMD(THF) level of theory and thermal correction to free energy obtained at B3LYP-GD3BJ/6-31g(d,p) level of theory.

	SCF	Gibbs
6(THF)	-3851.182629	0.579535
10	-4159.715883	0.679863
diphenylethylene (THF)	-540.968735	0.177586
HCl	-460.8404071	-0.011151
THF	-232.5650766	0.088548
10-ox	-4159.575952	0.680678
10-ox(THF)	-4392.143211	0.794936
10-ox(THF)-open	-4392.169592	0.792994
10-HCl	-4620.563998	0.686409
13	-4620.5758580	0.684945
14	-4620.653301	0.68641
10-H	-4160.154871	0.689476
10-open-H	-4160.154775	0.691568
10-H triplet	-4160.132516	0.685055
15	-3619.16163	0.474087
16	-4160.202815	0.685857
20	-3618.563766	0.464665
21	-4620.555255	0.688744
22	-4620.567873	0.687784
23	-4620.546675	0.68865
24	-4620.536946	0.68049
10-open-H-singlet broken symmetry	-4160.154773	0.691572
10-open-H-triplet	-4160.143053	0.686827
TS fragmentation from 10-ox(THF)-open	-4392.150313	0.791015
TS cycloreversion from 10-ox	-4159.54462	0.677671
TS oxidative addition HCl to 13	-4620.548324	0.682737
TS reductive elimination to 14	-4620.573594	0.684557
TS sigma metathesis from 10-HCl	-4620.543841	0.680155
TS metathesis 10-HCl	-4620.507061	0.679534
TS hydride insertion to 16	-4160.134176	0.688024
TS fragmentation from 10-open-H	-4160.129959	0.683876

Table S1. Single point energies obtained at the B3LYP-GD3BJ/DEF2TZVP/SMD(Toluene) level of theory and thermal correction to free energy obtained at B3LYP-GD3BJ/6-31g(d,p) level of theory.

	**SCF**	**Gibbs**
10	-4159.716093	0.679863
H2	-1.179332962	-0.001324
10-H2	-4160.895661	0.692436
11	-4160.941352	0.694819
17	-4160.871927	0.693776
TS H2 activation	-4160.881971	0.689472

Table S3. Single point energies obtained at the B3LYP-GD3BJ/DEF2TZVP/SMD(C_6H_6) level of theory and thermal correction to free energy obtained at B3LYP-GD3BJ/6-31g(d,p) level of theory.

	**SCF**	**Gibbs**
10	-4159.716749	0.679863
Phenylacetylene	-308.5455561	0.079101
10-eta2(C,H)alkyne	-4468.267275	0.778734
10-eta2(C,C)alkyne	-4468.273741	0.783643
12	-4468.325027	0.778044
18	-4468.278836	0.784666
19	-4468.311641	0.785101
TS sigma metathesis phenylacetylene	-4468.257978	0.773699
TS insertion phenylacetylene	-4468.253699	0.780782
TS cyclopropanation with phenylacetylene	-4468.237646	0.782485

6. Literature references

[1] M. Brookhart, B. Grant, A. F. Volpe, *Organometallics* **1992**, *11*, 3920–3922.

[2] M. L. G. Sansores-Paredes, S. van der Voort, M. Lutz, M. Moret, *Angew. Chem. Int. Ed.* **2021**, *60*, 26518–26522.

[3] M. L. G. Sansores-Paredes, M. Lutz, M. Moret, *Nat. Chem.* **2024**, *16*, 417-418.

[4] M. L. G. Sansores-Paredes, Tú T. T. Nguyen, M. Lutz, M. Moret, *Organometallics*, **2023**, *42, 23*, 3418-3427.

[5] M. L. Clapson, J. K. Kirkland, W. E. Piers, D. H. Ess, B. Gelfand, J. Bin Lin, *Organometallics* **2022**, *41*, 235–245.

[6] E. A. LaPierre, W. E. Piers, C. Gendy, *Dalton Trans.* **2018**, *47*, 16789–16797.

[7] C. C. Comanescu, M. Vyushkova, V. M. Iluc, *Chem. Sci.* **2015**, *6*, 4570-4579

[8] A. M. M. Schreurs, X. Xian, L. M. J. Kroon-Batenburg, *J. Appl. Crystallogr.* **2010**, *43*, 70–82.

[9] L. Krause, R. Herbst-Irmer, G. M. Sheldrick, D. Stalke, *J. Appl. Crystallogr.* **2015**, *48*, 3–10.

[10] G. M. Sheldrick, *Acta Crystallogr. Sect. A Found. Crystallogr.* **2015**, *71*, 3–8.

[11] G. M. Sheldrick, *Acta Crystallogr. Sect. C Struct. Chem.* **2015**, *71*, 3–8.

[12] A. L. Spek, *Acta Crystallogr. Sect. D Biol. Crystallogr.* **2009**, *65*, 148–155.

[13] R. Herbst-Irmer, G. M. Sheldrick, *Acta Crystallogr. Sect. B Struct. Sci.* **1998**, *54*, 443–449.

[14] G. M. Sheldrick (2014) SADABS and TWINABS. Universität Göttingen, Germany**.**

[15] M. J. Frisch, G. W. Trucks, H. B. Schlegel, G. E. Scuseria, M. A. Robb, J. R. Cheeseman, G. Scalmani, V. Barone, G. A. Petersson, H. Nakatsuji, X. Li, M. Caricato, A. V. Marenich, J. Bloino, B. G. Janesko, R. Gomperts, B. Mennucci, H. P. Hratchian, J. V. Ortiz, A. F. Izmaylov, J. L. Sonnenberg, D. Williams-Young, F. Ding, F. Lipparini, F. Egidi, J. Goings, B. Peng, A. Petrone, T. Henderson, D. Ranasinghe, V. G. Zakrzewski, J. Gao, N. Rega, G. Zheng, W. Liang, M. Hada, M. Ehara, K. Toyota, R. Fukuda, J. Hasegawa, M. Ishida, T. Nakajima, Y. Honda, O. Kitao, H. Nakai, T. Vreven, K. Throssell, J. A. Montgomery, Jr., J. E. Peralta, F. Ogliaro, M. J. Bearpark, J. J. Heyd, E. N. Brothers, K. N. Kudin, V. N. Staroverov, T. A. Keith, R. Kobayashi, J. Normand, K. Raghavachari, A. P. Rendell, J. C. Burant, S. S. Iyengar, J. Tomasi, M. Cossi, J. M. Millam, M. Klene, C. Adamo, R. Cammi, J. W. Ochterski, R. L. Martin, K. Morokuma, O. Farkas, J. B. Foresman, and D. J. Fox, **2016**, Gaussian 16, Revision C.01, Gaussian, Inc., Wallin.
